# Global variation in anastomosis and end colostomy formation following left‐sided colorectal resection

**DOI:** 10.1002/bjs5.50138

**Published:** 2019-02-28

**Authors:** GlobalSurg Collaborative, Writing group, James C Glasbey, Adewale O Adisa, Ainhoa Costas‐Chavarri, Ahmad U Qureshi, Jean C Allen‐Ingabire, Hosni Khairy Salem, Anyomih Theophilus Teddy Kojo, Stephen Tabiri, Dmitri Nepogodiev, Richard J Lilford, Ewen M Harrison, Thomas D Pinkney, Neil Smart, Aneel Bhangu, Patient representatives, Azmina Verjee, Emmy Runigamugabo, Statistical analysis, James Glasbey, Aneel Bhangu, Protocol development and project steering, Adesoji O Ademuyiwa, Adewale O Adisa, Maria Lorena Aguilera, Afnan Altamini, Philip Alexander, Sara W Al‐Saqqa, Giuliano Borda‐Luque, Jen Cornick, Ainhoa Costas‐Chavarri, Thomas M Drake, Stuart J Fergusson, J Edward Fitzgerald, James Glasbey, J.C Allen Ingabire, Lawani Ismaïl, Zahra Jaffry, Hosni Khairy Salem, Chetan Khatri, Andrew Kirby, Anyomih Theophilus Teddy Kojo, Marie Carmela Lapitan, Richard Lilford, Andre L Mihaljevic, Midhun Mohan, Dion Morton, Alphonse Zeta Mutabazi, Dmitri Nepogodiev, Faustin Ntirenganya, Riinu Ots, Francesco Pata, Thomas Pinkney, Tomas Poškus, Ahmad Uzair Qureshi, Antonio Ramos‐De la Medina, Sarah Rayne, Gustavo Recinos, Kjetil Søreide, Catherine A Shaw, Sebastian Shu, Richard Spence, Neil Smart, Stephen Tabiri, Richard Lilford, Dion Morton, Ewen M Harrison, Aneel Bhangu, National leads (GlobalSurg‐1), Chetan Khatri, Neel Gobin, Ana Vega Freitas, Nigel Hall, Sung‐Hee Kim, Ahmed Negida, Hosni Khairy, Zahra Jaffry, Stephen J Chapman, Alexis P Arnaud, Stephen Tabiri, Gustavo Recinos, Cutting Edge Manipal, Midhun Mohan, Radhian Amandito, Marwan Shawki, Michael Hanrahan, Francesco Pata, Justas Zilinskas, April Camilla Roslani, Cheng Chun Goh, Adesoji O Ademuyiwa, Gareth Irwin, Sebastian Shu, Laura Luque, Hunain Shiwani, Afnan Altamimi, Mohammed Ubaid Alsaggaf, Stuart J Fergusson, Richard Spence, Sarah Rayne, Jenifa Jeyakumar, Yucel Cengiz, Dmitri A Raptis, James C Glasbey, National leads (GlobalSurg‐2), Maria Marta Modolo, Dushyant Iyer, Sebastian King, Tom Arthur, Sayeda Nazmum Nahar, Ade Waterman, Lawani Ismaïl, Michael Walsh, Arnav Agarwal, Augusto Zani, Mohammed Firdouse, Tyler Rouse, Qinyang Liu, Juan Camilo Correa, Hosni Khairy Salem, Peep Talving, Mengistu Worku, Alexis Arnaud, Stephen Tabiri, Vassilis Kalles, Maria Lorena Aguilera, Gustavo Recinos, Basant Kumar, Sunil Kumar, Radhian Amandito, Roy Quek, Francesco Pata, Luca Ansaloni, Ahmed Altibi, Donatas Venskutonis, Justas Zilinskas, Tomas Poskus, John Whitaker, Vanessa Msosa, Yong Yong Tew, Alexia Farrugia, Elaine Borg, Antonio Ramos‐De la Medina, Zineb Bentounsi, Adesoji O Ademuyiwa, Kjetil Søreide, Tanzeela Gala, Ibrahim Al‐Slaibi, Haya Tahboub, Osaid H. Alser, Diego Romani, Sebestian Shu, Piotr Major, Aurel Mironescu, Matei Bratu, Amar Kourdouli, Aliyu Ndajiwo, Abdulaziz Altwijri, Mohammed Ubaid Alsaggaf, Ahmad Gudal, Al Faifi Jubran, Sam Seisay, Bettina Lieske, Sarah Rayne, Richard Spence, Irene Ortega, Jenifa Jeyakumar, Kithsiri J. Senanayake, Omar Abdulbagi, Yucel Cengiz, Dmitri Raptis, Yuksel Altinel, Chia Kong, Ella Teasdale, Gareth Irwin, Michael Stoddart, Rakan Kabariti, Sukrit Suresh, Katherine Gash, Ragavan Narayanan, Mayaba Maimbo, Local collaborators (GlobalSurg‐1), Claudio Fermani, Ruben Balmaceda, Maria Marta Modolo, Ewan Macdermid, Neel Gobin, Roxanne Chenn, Cheryl Ou Yong, Michael Edye, Martin Jarmin, Scott K D'amours, Dushyant Iyer, Daniel Youssef, Nicholas Phillips, Jason Brown, Robert George, Cherry Koh, Oliver Warren, Isaac Hanley, Marilla Dickfos, Clemens Nawara, Dietmar Öfner, Florian Primavesi, Ashrarur Rahman Mitul, Khalid Mahmud, Margub Hussain, Hafiz Hakim, Tapan Kumar, Antje Oosterkamp, Pamphile A Assouto, Ismail Lawani, Yacoubou Imorou Souaibou, Aung Kyaw Tun, Chean Leung Chong, Giridhar H Devadasar, Chean Leung Chong, Muhammad Rashid Minhas Qadir, Kyaw Phyo Aung, Lee Shi Yeo, Chean Leung Chong, Vanessa Dina Palomino Castillo, Monique Moron Munhoz, Gisele Moreira, Luiz Carlos Barros De Castro Segundo, Salim Anderson Khouri Ferreira, Maíra Cassa Careta, Stella Binna Kim, Alexandre Venancio De Sousa, Alyne Daltri Lazzarini Cury, Gustavo Peixoto Soares Miguel, Ana Vega Carreiro De Freitas, Barbara Pereira Silvestre Julia Guasti, Pinto Vianna, Carolina Oliveira Felipe, Luis Alberto Valente Laufer, Fernanda Altoe, Luana Ayres Da Silva, Marina Luiza Pimenta, Thiago Fernandes Giuriato, Paulo Alves Bezerra Morais, Jessica Souza Luiz Rafael Araujo, Juliana Menegussi, Marisa Leal, Caio Vinícius Barroso de Lima, Luiza Sarmento Tatagiba, Antônio Leal, Diogo Vinicius dos Santos, Gustavo Pereira Fraga, Romeo Lages Simoes, Simon Stock, Samuel Nigo, Juana Kabba, Tagang Ebogo Ngwa, James Brown, Sebastian King, Augusto Zani, Georges Azzie, Mohammed Firdouse, Sameer Kushwaha, Arnav Agarwal, Karen Bailey, Brian Cameron, Michael Livingston, Alexandre Horobjowsky, Dan L Deckelbaum, Tarek Razek, Boris Marinkovic, Eugenio Grasset, Nicole D'aguzan, Eugenio Grasset, Julio Jimenez, Roberto Macchiavello, Zhongtao Zhang, Wei Guo, Junyeong Oh, Fei Zheng, Irene Montes, Sebastian Sierra, Manuela Mendez, Maria Isabel Villegas, Maria Clara Mendoza Arango, Ivan Mendoza, Fred Alexander Naranjo Aristizã¡bal, Jaime Andres Montoya Botero, Victor Manuel Quintero Riaza, Jakeline Restrepo, Carlos Morales, Maria Clara Mendoza Arango, Herman Cruz, Alejandro Munera, Maria Clara Mendoza Arango, Robert Karlo, Edgar Domini, Jakov Mihanovic, Mihael Radic, Kresimir Zamarin, Nikica Pezelj, Manuel Hache‐Marliere, Sylvia Batista Lemaire, Ruben Rivas, Ahmed Khyrallh, Ahamed Hassan, Gamal Shimy, Mohamed A Baky Fahmy, Ayman Nabawi, Mohamed Elfil, Mohamed Ghoneem, Muhammad El‐Saied Ahmad Muhammad Gohar, Mohamed Asal, Mostafa Abdelkader, Mahmoud Gomah, Hayssam Rashwan, Mohamed Karkeet, Ahmed Gomaa, Amr Hasan, Ahmed Elgebaly, Omar Saleh, Ahmad Abdel Fattah, Abdullah Gouda, Abd Elrahman Elshafay, Abdalla Gharib, Ahmed Menshawy, Mohammed Hanafy, Abdullah Al‐Mallah, Mahmoud Abdulgawad, Mohamad Baheeg, Mohammed Alhendy, Ibrahim AbdelFattah, Abdalla Kenibar, Omar Osman, Mostafa Gemeah, Ahmed Mohammed, Abdalrahman Adel, Abdalla Gharib, Abdelrahman Mohammed, Abdelrahman Sayed, Mohamed Abozaid, Ahmed Hafez El‐Badri Kotb, Ali Amin Ahmed Ata, Mohammed Nasr, Abdelrahman Alkammash, Mohammed Saeed, Nader Abd El Hamid, Attia Mohamed Attia, Ahmed Abd El Galeel, Eslam Elbanby, Khalid Salah El‐Dien, Usama Hantour, Omar Alahmady, Billal Mansour, Amr Muhammad Elkorashy, Emad Mohamed Saeed Taha, Kholod Tarek Lasheen, Salma Said Elkolaly, Nehal Yosri Elsayed Abdel‐Wahab, Mahmoud Ahmed Fathi Abozyed, Ahmed Adel, Ahmed Moustafa Saeed, Gehad Samir El Sayed, Jehad Hassan Youssif, Soliman Magdy Ahmed, Nermeen Soubhy El‐Shahat, Abd El‐Rahman Hegazy Khedr, Abdelrhman Osama Elsebaaye, Mohamed Elzayat, Mohamed Abdelraheim, Ibrahim Elzayat, Mahmoud Warda, Khaled Naser El Deen, Abdelrhman Essam Elnemr, Omar Salah, Mohamed Abbas, Mona Rashad, Ibrahim Elzayyat, Dalia Hemeda, Gehad Tawfik, Mai Salama, Hazem Khaled, Mohamed Seisa, Kareem Elshaer, Abdelfatah Hussein, Mahmoud Elkhadrawi, Ahmed Mohamed Afifi, Osama Saadeldeen Ebrahim, Mahmoud Mohamed Metwally, Rowida Elmelegy, Diaa Moustafa Elbendary Elsawahly, Hisham Safa, Eman Nofal, Mohamed Elbermawy, Ahmed Abdelmotaleb Ghazy, Hisham Samih, Asmaa Abdelgelil, Sarah Abdelghany, Ahmed El Kholy, Metwally Aboraya, Fatma Elkady, Mahmoud Salma, Sarah Samy, Reem Fakher, Aya Aboarab, Ahmed Samir, Ahmed Sakr, Abdelrahman Haroun, Asmaa Abdel‐Rahman Al‐Aarag, Ahmed Elkholy, Sally Elshanwany, Esraa Ghanem, Ahmed Tammam, Ali Mohamed Hammad, Yousra El Shoura, Gehad El Ashal, Hosni Khairy, Sarah Antar, Sara Mehrez, Mahmoud Abdelshafy, Maha Gamal Mohamad Hamad, Mona Hosh, Emad Abdallah, Basma Magdy, Thuraya Alzayat, Elsayed Gamaly, Hossam Elfeki, Amany Abouzahra, Shereen Elsheikh, Fatimah I Elgendy, Fathia Abd El‐Salam, Osama Seifelnasr, Mohamed Ammar, Athar Eysa, Aliaa Sadek, Aliaa Gamal Toeema, Aly Nasr, Mohamed Abuseif, Hagar Zidan, Sara Abd Elmageed Barakat, Nadin Elsayed, Yasmin Abd Elrasoul, Ahmed Elkelany, Mohamed Sabry Ammar, Mennat‐Allah Mustafa, Yasmin Hegazy, Mohamed Etman, Samar Saad, Mahmoud Alrahawy, Ahmed Raslan, Mahmoud Morsi, Ahmed Rslan, Ahmed Sabry, Hager Elwakil, Heba Shaker, Hagar Zidan, Yasmin Abd‐Elrasoul, Ahmed Elkelany, Hussein El‐Kashef, Mohamed Shaalan, Areej Tarek, Ayman Elwan, Ahmed Ragab Nayel, Mostafa Seif, Ayman Elwan, Doaa Emadeldin, Mohamed Ali Ghonaim, Ahmad Almallah, Ahmed Fouad, Eman Adel Sayma, Ahmad Elbatahgy, Angham Solaiman El‐Ma'doul, Ahmed Mosad, Hager Tolba, Diaa Eldin Abdelazeem Amin Elsorogy, Hassan Ali Mostafa, Amira Atef Omar, Ola Sherief Abd El Hameed, Ahmed Lasheen, Yasser Abd El Salam, Ashraf Morsi, Mohammed Ismail, Hager Ahmed El‐badawy, Mohamed A Amer, Ahmed Elkelany, Ahmed Elkelany, Ahmed Sabry El‐Hamouly, Noura A. Attallah, Omnia Mosalum, Ahmed Afandy, Ahmed Mokhtar, Alaa Abouelnasr, Sara Ayad, Ramdan Shaker, Rokia Sakr, Ramadan Shaker, Mahmoud Amreia, Soaad Elsobky, Mohamed Mustafa, Ahmed Abo El Magd, Abeer Marey, Amr Tarek Hafez, Mohamed F Zalabia, Mohamed Moamen Mohamed, Amr Fadel, Emad Ali Ahmed, Ahmad Ali, Mohammad Ghassan Alwafai, Abdullah Dwydar, Sara Kharsa, Ehab Mamdouh, Hatem El‐Sheemy, Ibrahim AlYoussef, Abouelatta Khairy Aly, Ahmad Aldalaq, Ehab Alnawam, Dalia Alkhabbaz, Mahmoud Saad, Shady Hussein, Ahmed Abo Elazayem, Ahmed Meshref, Marwa Elashmawy, Mohammed Mousa, Ahmad Nashaat, Sara Ghanem, Zaynab M Elsayed, Aya Elwaey, Iman Elkadsh, Mariam Darweesh, Ahmed Mohameden, Mennaallah Hafez, Ahmed Badr, Assmaa Badwy, Mohamed Abd El Slam, Mohamed Elazoul, Safwat Al‐Nahrawi, Lotfy Eldamaty, Fathee Nada, Mohamed Ameen, Aya Hagar, Mohamed Elsehimy, Mohammad Aboraya, Hossam Dawoud, Shorouk El Mesery, Abeer El Gendy, Ahmed Abdelkareem, Ahmed Safwan Marey, Mostafa Allam, Sherif Shehata, Khaled Abozeid, Marwa Elshobary, Ahmed Fahiem, Sameh Sarsik, Amel Hashish, Mohamed Zidan, Mohamed Hashish, Shaimaa Aql, Abdelaziz Osman Abdelaziz Elhendawy, Mohamed Husseini, Esraa Kasem, Ahmed Gheith, Yasmin Elfouly, Ahmed Ragab Soliman, Yasmein Ibrahim, Nesma Elfouly, Ahmed Fawzy, Ahmed Hassan, Mohammad Rashid, Abdallah Salah Elsherbiny, Basem Sieda, Nermin M Badwi, Mohammed Mustafa Hassan Mohammed, Osama Mohamed, Mohammad Abdulkhalek Habeeb, Mengistu Worku, Nichole Starr, Semay Desta, Sahlu Wondimu, Nebyou Seyoum Abebe, Efeson Thomas, Frehun Ayele Asele, Daniel Dabessa, Nebiyou Seyoum Abebe, Abebe Bekele Zerihun, Panu Mentula, Ari Leppäniemi, Ville Sallinen, Aurelien Scalabre, Fernanda Frade, Sabine Irtan, Vivien Graffeille, Elodie Gaignard, Quentin Alimi, Quentin Alimi, Vivien Graffieille, Elodie Gaignard, Olivier Abbo, Sofia Mouttalib, Ourdia Bouali, Erik Hervieux, Yves Aigrain, Nathalie Botto, Alice Faure, Lucile Fievet, Nicoleta Panait, Emilie Eyssartier, Francoise Schmitt, Guillaume Podevin, Valentine Parent, Amandine Martin, Alexis Pierre Arnaud, Cecile Muller, Arnaud Bonnard, Matthieu Peycelon, Francis Abantanga, Kwaku Boakye‐Yiadom, Mohammed Bukari, Frank Owusu, Joseph Awuku‐Asabre, Stephen Tabiri, Lemuel Davies Bray, Dimitrios Lytras, Kyriakos Psarianos, Anastasia Bamicha, Eirini Kefalidi, Georgios Gemenetzis, Christos Dervenis, Nikolaos Gouvas, Christos Agalianos, Michail Kontos, Gregory Kouraklis, Dimitrios Karousos, Stylianos Germanos, Constantinos Marinos, Christos Anthoulakis, Nikolaos Nikoloudis, Nikolaos Mitroudis, Gustavo Recinos, Sergio Estupinian, Walter Forno, José René Arévalo Azmitia, Carla Cecilia Ramã­rez Cabrera, Romeo Guevara, Maria Aguilera, Napoleon Mendez, Cesar Augusto Azmitia Mendizabal, Pablo Ramazzini, Mario Contreras Urquizu, Fernando Tale, Rafael Soley, Emanuel Barrios, Emmanuel Barrios, Daniel Estuardo Marroquín Rodríguez, Carlos Iván Pérez Velásquez, Sara María Contreras Mérida, Francisco Regalado, Mario Lopez, Miguel Siguantay, Fong Yee Lam, Kylie Joan‐yi Szeto, Charing Cheuk Ling Szeto, Wing Sum Li, Kieran Ka Kei Li, Man Fung Leung, Tony Mak, Simon Ng, SS Prasad, Anand Kirishnan, Nidhi Gyanchandani, Bylapudi Seshu Kumar, Muthukumaran Rangarajan, Sriram Bhat, Anjana Sreedharan, S.V. Kinnera, Yella Reddy, Caranj Venugopal, Sunil Kumar, Abhishek Mittal, Shravan Nadkarni, Harish Neelamraju Lakshmi, Puneet Malik, Neel Limaye, Srinivas Pai, Pratik Jain, Monty Khajanchi, Savni Satoskar, Rajeev Satoskar, Abid Bin Mahamood, Eldaa Prisca Refianti Sutanto, Daniel Ardian Soeselo, Chintya Tedjaatmadja, Fitriana Nur Rahmawati, Radhian Amandito, Maria Mayasari, Ruqaya Kadhim Mohammed Jawad Al‐Hasani, Hasan Ismael Ibraheem Al‐Hameedi, Hasan Ismael Ibraheem, Israa Abdullah Aziz Al‐Azraqi, Lubna Sabeeh, Rahma Kamil, Marwan Shawki, Muwaffaq Mezeil Telfah, Amoudtha Rasendran, Jacqueline Sheehan, Robert Kerley, Caoimhe Normile, Richard William Gilbert, Jiheon Song, Mohamed Dablouk, Linnea Mauro, Mohammed Osman Dablouk, Michael Hanrahan, Paul Kielty, Eleanor Marks, Simon Gosling, Michelle Mccarthy, Amoudtha Rasendran, Diya Mirghani, Syed Altaf Naqvi, Chee Siong Wong, Siyi Chung, Reuban D'cruz, Ronan Cahill, Simon George Gosling, Michelle Mccarthy, Amoudtha Rasendran, Ciara Fahy, Jiheon Song, Michael Hanrahan, Diana Duarte Cadogan, Anna Powell, Richard Gilbert, Caroline Clifford, Caoimhe Normile, Aoife Driscoll, Stassen Paul, Chris Lee, Ross Bowe, William Hutch, Michael Hanrahan, Helen Mohan, Maeve O'neill, Kenneth Mealy, Piergiorgio Danelli, Andrea Bondurri, Anna Maffioli, Mario Pasini, Giacomo Pata, Stefano Roncali, Paolo Silvani, Michele Carlucci, Roberto Faccincani, Luigi Bonavina, Yuri Macchitella, Chiara Ceriani, Gregorio Tugnoli, Salomone Di Saverio, Khaled Khattab, Miguel Angel Paludi, Domenica Pata, Luigi Maria Cloro, Andrea Allegri, Luca Ansaloni, Federico Coccolini, Ezio Veronese, Luca Bortolasi, Alireza Hasheminia, Giacomo Nastri, Massimiliano Dal Canto, Stefano Cucumazzo, Francesco Pata, Angelo Benevento, Gaetano Tessera, Pier Paolo Grandinetti, Alessio Maniscalco, Giovanni Luca Lamanna, Luca Turati, Giovanni Sgroi, Emanuele Rausa, Roberta Villa, Michela Monteleone, David Merlini, Federico Coccolini, Luca Ansaloni, Andrea Allegri, Veronica Grassi, Roberto Cirocchi, Alban Cacurri, Hamza Waleed, Ahmed Diab, Fathi Elzowawi, Mantas Jokubauskas, Karolis Varkalys, Donatas Venskutonis, Robertas Pranevicius, Viktorija Ambrozeviciute, Simona Juciute, Austė Skardžiukaitė, Donatas Venskutonis, Saulius Bradulskis, Linas Urbanavicius, Aiste Austraite, Romualdas Riauka, Justas Zilinskas, Zilvinas Dambrauskas, Paulius Karumnas, Zigmantas Urniezius, Reda Zilinskiene, Anele Rudzenskaite, Ausrine Usaityte, Margarita Montrimaite, Nerijus Kaselis, Andrius Strazdas, Kristijonas Jokubonis, Kornelija Maceviciute, Virgilijus Beisa, Tomas Poskus, Kestutis Strupas, Erikas Laugzemys, Andrej Kolosov, Valdemaras Jotautas, Ignas Rakita, Saulius Mikalauskas, Darius Kazanavicius, Rokas Rackauskas, Kestutis Strupas, Tomas Poskus, Virgilijus Beisa, Ritauras Rakauskas, Egle Preckailaite, Ross Coomber, Kenneth Johnson, Jennifer Nowers, Dineshwary Periasammy, Afizah Salleh, Andre Das, Reuben Goh Ern Tze, Milaksh Nirumal Kumar, Nik Azim Nik Abdullah, Nik Ritza Kosai, Mustafa Taher, Reynu Rajan, Hoong Yin Chong, April Camilla Roslani, Cheng Chun Goh, Marija Agius, Elaine Borg, Maureen Bezzina, Roberta Bugeja, Martinique Vella‐Baldacchino, Andrew Spina, Josephine Psaila, Helene Francois‐Coridon, Cecilia Tolg, Jean‐Francois Colombani, Carmina Diaz‐Zorrilla, Antonio Ramos‐ De La Medina, Samantha Corro‐Diaz Gonzalez, Mário Jacobe, Domingos Mapasse, Elizabeth Snyder, Ramadan Oumer, Mohammed Osman, Aminu Mohammad, Lofty‐John Anyanwu, Abdulrahman Sheshe, Alaba Adesina, Olubukola Faturoti, Ogechukwu Taiwo, Muhammad Habib Ibrahim, Abdulrasheed A Nasir, Siyaka Itopa Suleiman, Adewale Adeniyi, Opeoluwa Adesanya, Ademola Adebanjo, Roland Osuoji, Kazeem Atobatele, Ayokunle Ogunyemi, Omolara Williams, Mobolaji Oludara, Olabode Oshodi, Adesoji Ademuyiwa, AbdulRazzaq Oluwagbemiga Lawal, Felix Alakaloko, Olumide Elebute, Adedapo Osinowo, Christopher Bode, Abidemi Adesuyi, Adesoji Tade, Adeleke Adekoya, Collins Nwokoro, Omobolaji O Ayandipo, Taiwo Akeem Lawal, Akinlabi E Ajao, Samuel Sani Ali, Babatunde Odeyemi, Samson Olori, Ademola Popoola, Ademola Adeyeye, James Adeniran, William J. Lossius, Ingemar Havemann, Kenneth Thorsen, Jon Kristian Narvestad, Kjetil Soreide, Trude Beate Wold, Linn Nymo, Mohammed Elsiddig, Manzoor Dar, Kamran Faisal Bhopal, Zainab Iftikhar, Muhammad Mohsin Furqan, Bakhtiar Nighat, Masood Jawaid, Abdul Khalique, Ahsan Zil‐E‐Ali, Anam Rashid, Hasnain Abbas Dharamshi, Tahira Naqvi, Ahmad Faraz, Abdul Wahid Anwar, Tahir Muhammad Yaseen, Ghina Shamim Shamsi, Ghina Shamsi, Tahir Yaseen, Wahid Anwer, Horacio Paredes Decoud, Omar Aguilera, Ismael Isaac Zelada Alvarez, Juan Marcelo Delgado, Gustavo Miguel Machain Vega, Helmut Alfredo Segovia Lohse, Wendy Leslie Messa Aguilar, Jose Antonio Cabala Chiong, Ana Cecilia Manchego Bautista, Eduardo Huaman, Sergio Zegarra, Rony Camacho, Jose María Vergara Celis, Diego Alonso Romani Pozo, José Hamasaki, Edilberto Temoche, Jaime Herrera‐Matta, Carla Pierina García Torres, Luis Miguel Alvarez Barreda, Ronald Renato Barrionuevo Ojeda, Octavio Garaycochea, Melanie Castro Mollo, Mitchelle Solange De Fã Tima Linares Delgado, Francisco Fujii, Ana Cecilia Manchego Bautista, Wendy Leslie Messa Aguilar, Jose Antonio Cabala Chiong, Susana Yrma Aranzabal Durand, Carlos Alejandro Arroyo Basto, Nelson Manuel Urbina Rojas, Sebastian Bernardo Shu Yip, Ana Lucia Contreras Vergara, Andrea Echevarria Rosas Moran, Giuliano Borda Luque, Manuel Rodriguez Castro, Ramon Alvarado Jaramillo, George Manrique Sila, Crislee Elizabeth Lopez, Mardelangel Zapata Ponze De Leon, Massiell Machaca, Ronald Coasaca Huaraya, Andy Arenas, Crislee López, Clara Milagros Herrera Puma, Wilfredo Pino, Christian Hinojosa, Melanie Zapata Ponze De Leon, Susan Limache, George Manrrique Sila, Layza‐Alejandra Mercado Rodriguez, Renato Melo, Jose Costa‐Maia, Nuno Muralha, Frederique Sauvat, Ionasc Dan, Mircea Hogea, Pandi Eduard, Razvan‐Matei Bratu, Mircea Beuran, Ionut‐Bogdan Diaconescu, Bogdan‐Valeriu Martian, Florin‐Mihail Iordache, Mihaela Vartic, Lucian Corneliu Vida, Liviu Iuliu Muntean, Aurel Sandu Mironescu, Vizir Jean Paul Nsengimana, Alice Niragire, Jean De La Croix Allen Ingabire, Eugene Niyirera, Nicola Zanini, Elio Jovine, Giovanni Landolfo, Ibrahim N. Alomar, Saleh A. Alnuqaydan, Abdulrahman M. Altwigry, Moayad Othman, Nohad Osman, Enas Alqahtani, Mohammed Alzahrani, Rifan Alyami, Emad Aljohani, Ibrahim Alhabli, Zaher Mikwar, Sultan Almuallem, Emad Aljohani, Rifan Alyami, Mohammed Alzahrani, Abrar Nawawi, Mohamad Bakhaidar, Ashraf A. Maghrabi, Mohammed Alsaggaf, Murad Aljiffry, Abdulmalik Altaf, Ahmad Khoja, Alaa Habeebullah, Nouf Akeel, Nashat Ghandora, Abdullah Almoflihi, Abdulmalik Huwait, Abeer Al‐shammari, Mashael Al‐Mousa, Masood Alghamdi, Walid Adham, Bandar Albeladi, Muayad Ahmed Alfarsi, Atif Mahdi, Saad Al Awwad, Afnan Altamimi, Thamer Nouh, Mazen Hassanain, Salman Aldhafeeri, Nawal Sadig, Osama Algohary, Mohannad Aledrisy, Ahmad Gudal, Ahmad Alrifaie, Mohammed AlRowais, Amani Althwainy, Alaa Shabkah, Uthman Alamoudi, Mawaddah Alrajraji, Basim Alghamdi, Saud Aljohani, Abdullah Daqeeq, Jubran J Al‐Faifi, Vicky Jennings, Nyawira Ngayu, Rachel Moore, Victor Kong, Hayden Kretzmann, Katie Connor, Daniel Nel, Colleen Sampson, Richard Spence, Eugenio Panieri, Sarah Rayne, Nosisa Sishuba, Myint Tun, Albert Mohale Mphatsoe, Jo‐Anne Carreira, Ella Teasdale, Mark Wagener, Stefan Botes, Danelo Du Plessis, Fernando Fernandez‐Bueno, Jose Aguilar‐Jimenez, Jose Andres Garcia‐Marin, Lorena Solar García, Luis Joaquín García Florez, Rubén Darío Arias Pacheco, Janet Pagnozzi, Jimy Harold Jara Quezada, Jose Luis Rodicio, German Minguez, Raquel Rodríguez‐Uría, Paul Ugalde, Camilo Lopez‐Arevalo, Luis Barneo, Jessica Patricia Gonzales Stuva, Irene Ortega‐Vazquez, Lorena Rodriguez, Norberto Herrera, Prasad Pitigala Arachchi, Wanigasekara Senanayake, Mudiyanselage Kithsiri Janakantha Senanayake, Lalith Asanka Jayasooriya Jayasooriya Arachchige, Sivasuriya Sivaganesh, Dulan Irusha Samaraweera, Vimalakanthan Thanusan, Ahmed Elgaili Khalid Musa, Reem Mohammed Hassan Balila, Mohamed Awad Elkarim Hamad Mohamed, Hussein Ali, Hagir Zain Elabdin, Alaa Hassan, Sefeldin Mahdi, Hala Ahmed, Sahar Abdoun Ishag Idris, Makki Elsayed, Mohammed Elsayed, Mohamed Mahmoud, Magnus Boijsen, Per‐Olof Lundgren, Ulf Gustafsson, Ali Kiasat, Fredrik Wogensen, Fredrik Wogensen, Emma Jurdell, Anders Thorell, Hildur Thorarinsdottir, Maria Utter, Sami Martin Sundstrom, Cecilia Wredberg, Ann Kjellin, Johanna Nyberg, Bjorn Frisk, Malin Sund, Linda Andersson, Ulf Gunnarsson, Yücel Cengiz, Sandra Ahlqvist, Ida Björklund, Hanna Royson, Per Weber, Hans‐Ivar Pahlsson, Eva Borin, Maria Hjertberg, Hanna Royson, Per Weber, Roger Schmid, Debora Schivo, Vasileios Despotidis, Stefan Breitenstein, Ralph F Staerkle, Erik Schadde, Fabian Deichsel, Alexandra Gerosa, Antonio Nocito, Dimitri Aristotle Raptis, Barbara Mijuskovic, Markus Zuber, Lukas Eisner, Swantje Kruspi, Katharina Beate Reinisch, Christin Schoewe, Allan Novak, Adrian F. Palma, Gerfried Teufelberger, Msafiri Kimaro, Rachel King, Ali Zeynel Abidin Balkan, Mehmet Gumar, Mehmet Ali Yavuz, Ufuk Karabacak, Gokhan Lap, Bahar Busra Ozkan, Bahar Busra Ozkan, Murat Karakahya, Ryan Adams, Robert Morton, Liam Henderson, Ruth Gratton, Keiran David Clement, Kate Yu‐Ching Chang, David Mcnish, Ryan Mcintosh, William Milligan, Brendan Skelly, Hannah Anderson‐Knight, Roger Lawther, Jemina Onimowo, Veereanna Shatkar, Shivanee Tharmalingam, Evelina Woin, Tessa Fautz, Oliver Ziff, Shiva Dindyal, Sam Arman, Shagorika Talukder, Sam Arman, Vijay Gadhvi, Shagorika Talukder, Luen Shaun Chew, Jonathan Heath, Natalie Blencowe, Sally Hallam, Katherine Gash, Gurdeep Singh Mannu, Dimitris‐Christos Zachariades, Ailsa Claire Snaith, Thusitha Sampath Hettiarachchi, Arjun Nesaratnam, James Wheeler, Darragh McCullagh, Joshua Michael Clements, Ata Khan, Foteini Koumpa, Christina Neophytou, Jessica Roth, Wai Cheong Soon, Mohammed Deputy, Ahmed Ahmed, Annelisse Ashton, Joe Vincent, Jack Almy, Taufiq Khan, John Lee Y Allen, Charlotte Jane Mcintyre, Dominic Charles Marshall, Mark Sykes, Nebil Behar, Harriet Jordan, Yaseen Rajjoub, Thomas Sherman, Timothy White, Anna Watts, Rohan Ardley, Tan Arulampalam, Apar Shah, Damien Brown, Emma Blower, Paul Sutton, Konstantinos Gasteratos, Dale Vimalachandran, Cathy Magee, Gareth Irwin, Andrew Mcguigan, Stephen Mcaleer, Clare Morgan, Sarah Braungart, Kirsten Lafferty, Peter Labib, Andrei Tanase, Clodagh Mangan, Lillian Reza, Lillian Reza, Andrei Tanase, Clodagh Mangan, Helen Woodward, Craig Gouldthorpe, Megan Turner, Jonathan R L Wild, Tom AM Malik, Victoria K Proctor, Kalon Hewage, James Davies, Andre Dubois, Sayed Sarwary, Ali Zardab, Alan Grant, Robert Mcintyre, Yogendra Praveen Mogan, Weiguang Ho, Bryon Frankie Hon Khi Chong, Shirish Tewari, Gemma Humm, Eriberto Farinella, Nigel J Hall, Naomi J Wright, Christina P Major, Thelma Xerri, Phoebe De Bono, Jasim Amin, Mustafa Farhad, John F. Camilleri‐Brennan, Andrew G N Robertson, Thelma Xerri, Joanna Swann, James Richards, Jasim Amin, Aijaz Jabbar, Phoebe De Bono, Myranda Attard, Hannah Burns, Euan Macdonald, Matthew Baldacchino, Jennifer Skehan, Julian Camilleri‐Brennan, Tom Falconer Hall, Madelaine Gimzewska, Greta Mclachlan, Jamie Shah, James Giles, Selina Chiu, Beatrix Weber, Selina Man Yeng Chiu, Saskia Highcock, Maleeha Hassan, William Beasley, Apostolos Vlachogiorgos, Stephen Dias, Geta Maharaj, Rosie Mcdonald, Alisdair Macdonald, Paul Witherspoon, Alan Baird, Panchali Sarmah, Nikki Green, Haney Youssef, Kate Cross, Clare M Rees, Bernard Van Duren, Emma Upchurch, Khurram Khan, Haytham Abudeeb, Ahmed Hammad, Sharad Karandikar, Doug Bowley, Ahmed Karim, Witold Chachulski, Liam Richardson, Giles Dawnay, Ben Thompson, Ajayesh Mistry, Aneel Bhangu, Millika Ghetia, Sudipta Roy, Ossama Al‐Obaedi, Millika Ghetia, Kaustuv Das, Ash Prabhudesai, DM Cocker, Jessica Juliana Tan, Robert Tyler, Filippo Di Franco, Shruti Ayyar, Sayinthen Vivekanantham, Shyam Gokani, Michael Gillespie, Katrin Gudlaugsdottir, Theodore Pezas, Chelise Currow, Matthew Young‐Han Kim, Amerdip Birring, Joanne Edwards, Ased Ali, Suparna Das, Madan Jha, Kieran Atkinson, Joshua Luck, Thomas Fozard, Michael Puttick, Yahya Salama, Rohi Shah, Ahmad Aboelkassem Ibrahem, Hamdi Ebdewi, Gianpiero Gravante, Saleem El‐Rabaa, Henry Nnajiuba, Rebecca Allott, Aman Bhargava, Zoe Chan, Zaffar Hassan, Misty Makinde, David Hemingway, Ramzana Dean, Alexander Boddy, Ahmed Aber, Vijay Patel, Jehangirshaw Parakh, Sunil Parthiban, Harmony Kaur Ubhi, Simon‐Peter Hosein, Simon Ward, Kamran Malik, Leifa Jennings, Tom Newton, Mirna Alkhouri, Min Kyu Kang, Christopher Houlden, Jonathan Barry, Imtanan Raza, Alistair Farquharson, Sanjeet Bhattacharya, William Milligan, Kate Chang, Liam Henderson, Michael S J Wilson, Yan Ning Neo, Ibrahim Ibrahim, Emily Chan, Fraser S Peck, Pei J Lim, Alexander S North, Rebecca Blundell, Adam Williamson, Dina Fouad, Ashish Minocha, Kathryn Mccarthy, Emma Court, Alice Chambers, Jenna Yee, Ji Chung Tham, Ceri Beaton, Una Walsh, Joseph Lockey, Salman Bokhari, Lara Howells, Megan Griffiths, Laura Yallop, Shailinder Singh, Omar Nasher, Paul Jackson, Michael Puttick, Joshua Luck, Thomas Fozard, Abdul Muiz Shariffuddin, Weng Chee Ho, Michael Sj Wilson, Gurpreet Pabla, Saed Ramzi, Shady Zeidan, Jennifer Doughty, Sidhartha Sinha, Ross Davenport, Jason Lewis, Leo Duffy, Elizabeth Mcaleer, Eleanor Williams, Robin Som, Omar Javed, Matthew Boal, Nicola Harrison, Habib Tafazal, Omar Javed, Tom Brogden, Dmitri Nepogodiev, Ewen Griffiths, Rhalumi Daniel Obute, Thomas E Glover, David J Clark, Mohamed Boshnaq, Mansoor Akhtar, Pascale Capleton, Samer Doughan, Mohamed Rabie, Ismail Mohamed, Duncan Samuel, Lauren Dickson, Matthew Kennedy, Eleanor Dempster, Emma Brown, Natalie Maple, Eimear Monaghan, Bernhard Wolf, Alicia Garland, Arthur Mcphee, David Anderson, Robert Anderson, Sarah Hassan, Paul Sutton, Dave Smith, Jonathan Lund, Catherine Boereboom, Jennifer Murphy, Gillian Tierney, Samson Tou, Eleanor Franziska Zimmermann, Neil James Smart, Andrea Marie Warwick, Theodora Stasinou, Ian Daniels, Kim Findlay‐Cooper, Stefan Mitrasinovic, Swayamjyoti Ray, Massimo Varcada, Rovan D'Souza, Sharif Omara, Matthew Spurr, Lucienne Parkinson, Anthony Hanks, Jennifer Ma, Emily Abington, Meera Ramcharn, Gethin Williams, Joseph Winstanley, Ewan D. Kennedy, Emily NW Yeung, Stuart J Fergusson, Catrin Jones, Stephen O'neill, Shujing Jane Lim, Ignatius Liew, Hari Nair, Cameron Fairfield, Julia Oh, Samantha Koh, Andrew Wilson, Catherine Fairfield, Delran Anandkumar, Ashok Kirupagaran, Timothy F Jones, Hew Dt Torrance, Alexander J Fowler, Charmilie Chandrakumar, Priyank Patel, Syed Faaz Ashraf, Sonam M. Lakhani, Aaron Lawson Mclean, Sonia Basson, Jeremy Batt, Catriona Bowman, Michael Stoddart, Natasha Benons, Clare Mason, Rebecca Harrison, John Quayle, Tom Barker, Virginia Summerour, Edward Harper, Caroline Smith, Matthew Hampton, Sophie K Pitt, Alex E Ward, Timothy O'Connor, Emily G Heywood, Thomas M Drake, Abeed Chowdhury, Sina Hossaini, Nicholas Fs Watson, Doug Mckechnie, Ayaan Farah, Anita Chun, Hoey Koh, Grace Lim, Graham Sunderland, Laura Gould, Alice Chambers, P C Munipalle, H Rooney, D R L Browning, Bernadette Pereira, Kristof Nemeth, Emily Decker, Stefano Giuliani, Aly Shalaby, Shafaque Shaikh, Chern Yan Tan, Ebrahim Y A Palkhi, Aleksandra Szczap, Swathikan Chidambaram, Chee Yang Chen, Kavian Kulasabanathan, Srishti Chhabra, Elisabeth Kostov, Philippe Harbord, James Barnacle, Madan Mohan Palliyil, Mina Zikry, Johnathan Porter, Charef Raslan, Mohammed Saeed, Shazia Hafiz, Niksa Soltani, Katie Baillie, Priyanka Singh, Shailee Sheth, Kishen Patel, Mahry Khalili, Jeesoo Choi, Matthew Benger, Lucy Marples, Alastair Macfarlane, Ramesh Thurairaja, Tamsin Boyce, Harriet Whewell, Elin Jones, Francesca Th'ng, Nichola Robertson, Ahmad Mirza, Haroon Saeed, Simon Galloway, Gia Elena, Mohammad Afzal, Mohamed Zakir, Peter Sodde, Charles Hand, Aiesha Sriram, Tamsyn Clark, Patrick Holton, Amy Livesey, Yashashwi Sinha, Fahad Mujtaba Iqbal, Indervir Singh Bharj, Adriana Rotundo, Cara Jenvey, Robert Slade, David Golding, Samuel Haines, Ali Adel Ne'ma Abdullah, Thomas W Tilston, Dafydd Loughran, Danielle Donoghue, Lorenzo Giacci, Mohamed Ashur Sherif, Peter Harrison, Alethea Tang, Deevia Kotecha, Mohamed Elshaer, Tomas Urbonas, Amjid Riaz, Annie Chapman, Parisha Acharya, Joseph Shalhoub, Cathleen Grossart, David McMorran, Makhosini Mlotshwa, William Hawkins, Sofronis Loizides, Kandaswamy Krishna, Melanie Orchard, Chik Wai Ho, Peter Thomson, Shahab Khan, Fiona Taylor, Jalak Shukla, Emma Elizabeth Howie, Linda Macdonald, Olusegun Komolafe, Neil Mcintyre, James Cragg, Jody Parker, Duncan Stewart, Luke Lintin, Julia Tracy, Tahir Farooq, George Molina, Haytham Kaafarani, Laura Luque, Robel Beyene, Jack Sava, Mark Scott, Mamta Swaroop, Raelene Kennedy, Ijeoma A Azodo, Daithi Heffernan, Tristen Chun, Andrew Stephen, Melanie Sion, Michael S. Weinstein, Viren Punja, Nikolay Bugaev, Monica Goodstein, Shadi Razmdjou, Eric Etchill, Juan Carlos Puyana, Matthew Kesinger, Lena Napolitano, Kathleen To, Mark Hemmila, Oliver Todd, Edward Jenner, Ellen Hoogakker, Local collaborators (GlobalSurg‐2), Besmir Grizhja, Shpetim Ymeri, Gezim Galiqi, Roberto Klappenbach, Diego Antezana, Alvaro Enrique Mendoza Beleño, Cecilia Costa, Belen Sanchez, Susan Aviles, Maria Marta Modolo, Claudio Gabriel Fermani, Rubén Balmaceda, Santiago Villalobos, Juan Manuel Carmona, Daniel Hamill, Peter Deutschmann, Simone Sandler, Daniel Cox, Ram Nataraja, Claire Sharpin, Damir Ljuhar, Demi Gray, Morgan Haines, Dush Iyer, Nithya Niranjan, Scott D'Amours, Morvarid Ashtari, Helena Franco, Ashrarur Rahman Mitul, Sabbir Karim, Nowrin F. Aman, Mahnuma Mahfuz Estee, Umme Salma, Joyeta Razzaque, Tasnia Hamid Kanta, Sayeeda Aktar Tori, Shadid Alamin, Swapnil Roy, Shadid Al Amin, Rezaul Karim, Muhtarima Haque, Amreen Faruq, Farhana Iftekhar, Margaret O'Shea, Greg Padmore, Ramesh Jonnalagadda, Andrey Litvin, Aliaksandr Filatau, Dzmitry Paulouski, Maryna Shubianok, Tatsiana Shachykava, Dzianis Khokha, Vladimir Khokha, Fernande Djivoh, Lawani Ismaïl, Francis Dossou, Djifid Morel Seto, Dansou Gaspard Gbessi, Bruno Noukpozounkou, Yacoubou Imorou Souaibou, Kpèmahouton René Keke, Fred Hodonou, Ernest Yemalin Stephane Ahounou, Thierry Alihonou, Max Dénakpo, Germain Ahlonsou, Alemayehu Ginbo Bedada, Carlos Nsengiyumva, Sandrine Kwizera, Venerand Barendegere, Philip Choi, Simon Stock, Luai Jamal, Mohammed Firdouse, Augusto Zani, Georges Azzie, Sameer Kushwaha, Arnav Agarwal, Tzu‐Ling Chen, Chingwan Yip, Irene Montes, Felipe Zapata, Sebastian Sierra, Maria Isabel Villegas Lanau, Maria Clara Mendoza Arango, Ivan Mendoza Restrepo, Sebastian Sierra, Ruben Santiago Restrepo Giraldo, Maria Clara Mendoza Arango, Edgar Domini, Robert Karlo, Jakov Mihanovic, Mohamed Youssef, Hossam Elfeki, Waleed Thabet, Aly Sanad, Gehad Tawfik, Ahmed Zaki, Noran Abdel‐Hameed, Mohamed Mostafa, Muhammad Fathi Waleed Omar, Ahmed Ghanem, Emad Abdallah, Adel Denewar, Eman Emara, Eman Rashad, Ahmad Sakr, Rehab Elashry, Sameh Emile, Toqa Khafagy, Sara Elhamouly, Arwa Elfarargy, Amna Mamdouh Mohamed, Ghada Saied Nagy, Abeer Esam, Eman Elwy, Aya Hammad, Salwa Khallaf, Eman Ibrahim, Ahmed Saidbadr, Ahmed Moustafa, Amany Eldosouky Mohammed, Mohammed Elgheriany, Eman Abdelmageed, Eman Abd Al Raouf, Esraa Samir Elbanby, Maha Elmasry, Mahitab Morsy Farahat, Eman Yahya Mansor, Eman Magdy Hegazy, Esraa Gamal, Heba Gamal, Hend Kandil, Doaa Maher Abdelrouf, Mohamed Moaty, Dina Gamal, Nada El‐Sagheer, Mohamed Salah, Salma Magdy, Asmaa Salah, Ahmed Essam, Ahmed Ali, Mahmoud Badawy, Sara Ahmed, Mazed Mohamed, Abdelrahman Assal, Mohamed Sleem, Mai Ebidy, Aly Abd Elrazek, Diaaaldin Zahran, Nourhan Adam, Mohamed Nazir, Adel B Hassanein, Ahmed Ismail, Amira Elsawy, Rana Mamdouh, Mohamed Mabrouk, Lopna Ahmed Mohamed Ahmed, Mohamed Hassab Alnaby, Eman Magdy, Manar Abd‐Elmawla, Marwan Fahim, Bassant Mowafy, Moustafa Ibrahim Mahmoud, Meran Allam, Muhammad Alkelani, Noran Halim El Gendy, Mariam Saad Aboul‐Naga, Reham Alaa El‐Din, Alyaa Halim Elgendy, Mohamed Ismail, Mahmoud Shalaby, Aya Adel Elsharkawy, Mahmoud Elsayed Moghazy, Khaled Hesham Elbisomy, Hend Adel Gawad Shakshouk, Mohamed Fouad Hamed, Mai Mohamed Ebidy, Mostafa Abdelkader, Mohamed Karkeet, Hayam Ahmed, Israa Adel, Mohammad Elsayed Omar, Mohamed Ibrahim, Omar Ghoneim, Omar Hesham, Shimaa Gamal, Karim Hilal, Omar Arafa, Sawsan Adel Awad, Menatalla Salem, Fawzia Abdellatif Elsherif, Nourhan Elsabbagh, Moustafa R. Aboelsoud, Ahmed Hossam Eldin Fouad Rida, Amr Hossameldin, Ethar Hany, Yomna Hosny Asar, Nourhan Anwar, Mohamed Gadelkarim, Samar Abdelhady, Eman Mohamed Morshedy, Reham Saad, Nourhan Soliman, Mahmoud Salama, Eslam Ezzat, Arwa Mohamed, Arwa Ibrahim, Alaa Fergany, Sara Mohammed, Aya Reda, Yomna Allam, Hanan Adel Saad, Afnan Abdelfatah, Aya Mohamed Fathy, Ahmed El‐Sehily, Esraa Abdalmageed Kasem, Ahmed Tarek Abdelbaset Hassan, Ahmed Rabeih Mohammed, Abdalla Gamal Saad, Yasmin Elfouly, Nesma Elfouly, Arij Ibrahim, Amr Hassaan, Mohammed Mustafa Mohammed, Ghada Elhoseny, Mohamed Magdy, Esraa Abd Elkhalek, Yehia Zakaria, Tarek Ezzat, Ali Abo El Dahab, Mohamed Kelany, Sara Arafa, Osama Mokhtar Mohamed Hassan, Nermin Mohamed Badwi, Ahmad Saber Sleem, Hussien Ahmed, Kholoud Abdelbadeai, Mohamed Abozed Abdullah, Muhammad Amsyar Auni Lokman, Suraya Bahar, Anan Rady Abdelazeam, Abdelrahman Adelshone, Muhammad Bin Hasnan, Athirah Zulkifli, Siti Nur Alia Kamarulzamil, Abdelaziz Elhendawy, Aliang Latif, Ahmad Bin Adnan, Shahadatul Shaharuddin, Aminah Hanum Haji Abdul Majid, Mahmoud Amreia, Dina Al‐Marakby, Mahmoud Salma, Mohamad Jeffrey Bin Ismail, Elissa Rifhan Mohd Basir, Citra Dewi Mohd Ali, Aya Yehia Ata, Maha Nasr, Asmaa Rezq, Ahmed Sheta, Sherif Tariq, Abd Elkhalek Sallam, Abdelrhman KZ Darwish, Sohaila Elmihy, Shady Elhadry, Ahmed Farag, Haidar Hajeh, Abdelaziz Abdelaal, Amro Aglan, Ahmed Zohair, Mahitab Essam, Omar Moussa, Esraa El‐Gizawy, Mostafa Samy, Safia Ali, Esraa Elhalawany, Ahmed Ata, Mohamed El Halawany, Mohamed Nashat, Samar Soliman, Alaa Elazab, Mostada Samy, Mohamed A Abdelaziz, Khaled Ibrahim, Ahmed mohamed Ibrahim, Ammar Gado, Usama Hantour, Esraa Alm Eldeen, Mohamed Reda loaloa, Arwa Abouzaid, Mostafa Ahmed Bahaa Eldin, Eman Hashad, Fathy Sroor, Doaa Gamil, Eman Mahmoud Abdulhakeem, Mahmoud Zakaria, Fawzy Mohamed, Marwan Abubakr, Elsayed Ali, Hesham Magdy, Menna Tallah Ramadan, Mohamed Abdelaty Mohamed, Salma Mansour, Hager Abdul Aziz Amin, Ahmed Rabie Mohamed, Mahmoud Saami, Nada Ahmed Reda Elsayed, Adham Tarek, Sabry Mohy Eldeen Mahmoud, Islam Magdy El Sayed, Amira Reda, Martina Yusuf Shawky, Mohammed Mousa Salem, Shahinaz Alaa El‐Din, Noha Abdullah Soliman, Muhammed Talaat, Shahinaz Alaael‐Dein, Ahmed Abd Elmoen Elhusseiny, Noha Abdullah, Mohammed Elshaar, Aya AbdelFatah Ibraheem, Hager Abdulaziz, Mohammed Kamal Ismail, Mona Hamdy Madkor, Mohamed Abdelaty, Sara Mahmoud Abdel‐Kader, Osama Mohamed Salah, Mahmoud Eldafrawy, Ahmed Zaki Eldeeb, Mostafa Mahmoud Eid, Attia Attia, Khalid Salah El‐Dien, Ayman Shwky, Mohamed Adel Badenjki, Abdelrahman Soliman, Samaa Mahmoud Al Attar, Farrag Sayed, Fahd Abdel Sabour, Mohammed G. Azizeldine, Muhammad Shawqi, Abdullah Hashim, Ahmed Aamer, Ahmed Mahmoud Abdelraouf, Mahmoud Abdelshakour, Amal Ibrahim, Basma Mahmoud, Mohamed Ali Mahmoud, Mostafa Qenawy, Ahmed M. Rashed, Ahmed Dahy, Marwa Sayed, Ahmed W. Shamsedine, Bakeer Mohamed, Ahmad Hasan, Mahmoud M. Saad, Khalil Abdul Bassit, Nadia Khalid Abd El‐Latif, Nada Elzahed, Ahmed El Kashash, Nada Mohamed Bekhet, Sarah Hafez, Ahmed Gad, Mahmoud Elkhadragy Maher, Ahmed Abd Elsameea, Mohamed Hafez, Ahmad Sabe, Ataa Ahmed, Ahmed Shahine, Khaled Dawood, Shireen Gaafar, Reem Husseiny, Omnia Aboelmagd, Ahmed Soliman, Nourhan Mesbah, Hossam Emadeldin, Amgad Al Meligy, Amira Hassan Bekhet, Doaa Hasan, Khaled Alhady, Ahmad Khaled Sabe, Mahmoud A. Elnajjar, Majed Aboelella, Ward Hamsho, Ihab Hassan, Hala Saad, Galaleldin Abdelazim, Hend Mahmoud, Noha Wael, Ahmedali M Kandil, Ahmed Magdy, Shimaa Said Elkholy, Badr Eldin Adel, Kareem Dabbour, Saged Elsherbiney, Omar Mattar, Abdulshafi Khaled Abdrabou, Mohammed Yahia Mohamed Aly, Abdelrahman Geuoshy, Ahmedglal Elnagar, Saraibrahim Ahmed, Ibrahem Abdelmotaleb, Amr Ahmed Saleh, Manar Saeed, Shady Mahmoud, Badreldin Adel Tawfik, Samar Adel Ismail, Esraay Zakaria, Mariam O. Gad, Mohamed Salah Elhelbawy, Monica Bassem, Noha Maraie, Nourhan Medhat Elhadary, Nourhan Semeda, Shaza Rabie Mohamed, Hesham Mohammed Bakry, AA Essam, Dina Tarek, Khlood Ashour, Alaa Elhadad, Abdulrahman Abdel‐Aty, Ibrahim Rakha, Sara Mamdouh Matter, Rasha Abdelhamed, Omar Abdelkader, Ayat Hassaan, Yasmin Soliman, Amna Mohamed, Sara Ghanem, Sara Amr Mohamed Farouk, Eman Mohamed Ibrahim, Esraa El‐Taher, Merna Mostafa, Mohamed Fawzy Mahrous Badr, Rofida Elsemelawy, Aya El‐Sawy, Ahmad Bakr, Ahmad Abdel Razaq Al Rafati, Sten Saar, Arvo Reinsoo, Peep Talving, Nebyou Seyoum, Tewodros Worku, Agazi Fitsum, Matti Tolonen, Ari Leppäniemi, Ville Sallinen, Benoît Parmentier, Matthieu Peycelon, Sabine Irtan, Sabrina Dardenne, Elsa Robert, Betty Maillot, Etienne Courboin, Alexis Pierre Arnaud, Juliette Hascoet, Olivier Abbo, Amir Ait Kaci, Thomas Prudhomme, Quentin Ballouhey, Céline Grosos, Laurent Fourcade, Tolg Cecilia, Colombani Jean‐Francois, Francois‐Coridon Helene, Xavier Delforge, Elodie Haraux, Bertrand Dousset, Roberto Schiavone, Sebastien Gaujoux, Jean‐Baptiste Marret, Aurore Haffreingue, Julien Rod, Mariette Renaux‐Petel, Jean‐François Lecompte, Jean Bréaud, Pauline Gastaldi, Chouikh Taieb, Raquillet Claire, Echaieb Anis, Nasir Bustangi, Manuel Lopez, Aurelien Scalabre, Maria Giovanna Grella, Aurora Mariani, Guillaume Podevin, Françoise Schmitt, Erik Hervieux, Aline Broch, Cecile Muller, Stephen Tabiri, Anyomih Theophilus Teddy Kojo, Dickson Bandoh, Francis Abantanga, Martin Kyereh, Hamza Asumah, Eric Kofi Appiah, Paul Wondoh, Adam Gyedu, Charles Dally, Kwabena Agbedinu, Michael Amoah, Abiboye Yifieyeh, Frank Owusu, Mabel Amoako‐Boateng, Makafui Dayie, Richmond Hagan, Sam Debrah, Micheal Ohene‐Yeboah, Joe‐Nat Clegg‐Lampety, Victor Etwire, Jonathan Dakubo, Samuel Essoun, William Bonney, Hope Glover‐Addy, Samuel Osei‐Nketiah, Joachim Amoako, Niiarmah Adu‐Aryee, William Appeadu‐Mensah, Antoinette Bediako‐Bowan, Florence Dedey, Mattew Ekow, Emmanuel Akatibo, Musah Yakubu, Hope Edem Kofi Kordorwu, Kwasi Asare‐Bediako, Enoch Tackie, Kenneth Aaniana, Emmanuel Acquah, Richard Opoku‐Agyeman, Anthony Avoka, Kwasi Kusi, Kwame Maison, Frank Enoch Gyamfi, Gandau Naa Barnabas, Saiba Abdul‐Latif, Philip Taah Amoako, Anthony Davor, Victor Dassah, Enoch Dagoe, Prince Kwakyeafriyie, Elliot Akoto, Eric Ackom, Ekow Mensah, Ebenezer Takyi Atkins, Christian Lari Coompson, Nikolaos Ivros, Christoforos Ferousis, Vasileios Kalles, Christos Agalianos, Ioannis Kyriazanos, Christos Barkolias, Angelos Tselos, Georgios Tzikos, Evangelos Voulgaris, Dimitrios Lytras, Athanasia Bamicha, Kyriakos Psarianos, Anastasios Stefanopoulos, Ioannis Patoulias, Dimitrios Sfougaris, Ioannis Valioulis, Dimitrios Balalis, Dimitrios Korkolis, Dimitrios K Manatakis, Georgios Kyrou, Georgios Karabelias, Iason‐Antonios Papaskarlatos, Kolonia Konstantina, Nikolaos Zampitis, Stylianos Germanos, Aspasia Papailia, Theodosios Theodosopoulos, Georgios Gkiokas, Magdalini Mitroudi, Christina Panteli, Thomas Feidantsis, Konstantinos Farmakis, Dimitrios Kyziridis, Orestis Ioannidis, Styliani Parpoudi, Georgios Gemenetzis, Stavros Parasyris, Christos Anthoulakis, Nikolaos Nikoloudis, Michail Margaritis, Maria‐Lorena Aguilera‐Arevalo, Otto Coyoy‐Gaitan, Javier Rosales, Luis Tale, Rafael Soley, Emmanuel Barrios, Servio Tulio Torres Rodriguez, Carlos Paz Galvez, Danilo Herrera Cruz, Guillermo Sanchez Rosenberg, Alejandro Matheu, David Monterroso Cohen, Marie Paul, Angeline Charles, Justin Chak Yiu Lam, Man Hon Andrew Yeung, Chi Ying Jacquelyn Fok, Ka Hin Gabriel Li, Anthony Chuk‐Him Lai, Yuk Hong Eric Cheung, Hong Yee Wong, Ka Wai Leung, Tien Seng Bryan Lee, Wai Him Lam, Weihei Dao, Stephanie Hiu‐wai Kwok, Tsz‐Yan Katie Chan, Yung Kok Ng, TWC Mak, Qinyang Liu, Chi Chung Foo, James Yang, Basant Kumar, Ankur Bhatnagar, Vijaid Upadhyaya, Sunil Kumar, Uday Muddebihal, Wasim Dar, KC Janardha, Philip Alexander, Neerav Aruldas, Fidelis Jacklyn Adella, Anthonius Santoso Rulie, Ferdy Iskandar, Jonny Setiawan, Cicilia Viany Evajelista, Hani Natalie, Arlindawati Suyadi, Rudy Gunawan, Herlin Karismaningtyas, Lusi Padma Sulistianingsih Mata, Ferry Fitriya Ayu Andika, Afifatun Hasanah, T Ariani Widiastini, Nurlaila Ayu Purwaningsih, Annisa Dewi Fitriana Mukin, Dina Faizatur Rahmah, Hazmi Dwinanda Nurqistan, Hasbi Maulana Arsyad, Novia Adhitama, Wifanto Saditya Jeo, Nathania Sutandi, Audrey Clarissa, Phebe Anggita Gultom, Matthew Billy, Andreass Haloho, Radhian Amandito, Nadya Johanna, Felix Lee, Radin Mohd Nurrahman Radin Dorani, Martha Glynn, Mohammad Alherz, Wennweoi Goh, Haaris A. Shiwani, Lorraine Sproule, Kevin C. Conlon, Miklosh Bala, Asaf Kedar, Luca Turati, Federica Bianco, Francesca Steccanella, Gaetano Gallo, Mario Trompetto, Giuseppe Clerico, Matteo Papandrea, Giuseppe Sammarco, Rosario Sacco, Angelo Benevento, Francesco Pata, Luisa Giavarini, Mariano Cesare Giglio, Luigi Bucci, Gianluca Pagano, Viviana Sollazzo, Roberto Peltrini, Gaetano Luglio, Arianna Birindelli, Salomone Di Saverio, Gregorio Tugnoli, Miguel Angel Paludi, Pietro Mingrone, Domenica Pata, Francesco Selvaggi, Lucio Selvaggi, Gianluca Pellino, Natale Di Martino, Gianluca Curletti, Paolo Aonzo, Raffaele Galleano, Stefano Berti, Elisa Francone, Silvia Boni, Laura Lorenzon, Annalisa lo Conte, Genoveffa Balducci, Gianmaria Confalonieri, Giovanni Pesenti, Laura Gavagna, Giorgio Vasquez, Simone Targa, Savino Occhionorelli, Dario Andreotti, Giacomo Pata, Andrea Armellini, Deborah Chiesa, Fabrizio Aquilino, Nicola Chetta, Arcangelo Picciariello, Mohamed Abdelkhalek, Andrea Belli, Silvia De Franciscis, Annamaria Bigaran, Alessandro Favero, Stefano M.M Basso, Paola Salusso, Martina Perino, Sylvie Mochet, Diego Sasia, Francesco Riente, Marco Migliore, David Merlini, Silvia Basilicò, Carlo Corbellini, Veronica Lazzari, Yuri Macchitella, Luigi Bonavina, Daniele Angelieri, Diego Coletta, Federica Falaschi, Marco Catani, Claudia Reali, Mariastella Malavenda, Celeste Del Basso, Sergio Ribaldi, Massimo Coletti, Andrea Natili, Norma Depalma, Immacolata Iannone, Angelo Antoniozzi, Davide Rossi, Daniele Gui, Gerardo Perrotta, Matteo Ripa, Francesco Ruben Giardino, Maurizio Foco, Erika Vicario, Federico Coccolini, Luca Ansaloni, Gabriela Elisa Nita, Nicoletta Leone, Andrea Bondurri, Anna Maffioli, Andrea Simioni, Davide De Boni, Sandro Pasquali, Elena Goldin, Elena Vendramin, Eleonora Ciccioli, Umberto Tedeschi, Luca Bortolasi, Paola Violi, Tommaso Campagnaro, Simone Conci, Giovanni Lazzari, Calogero Iacono, Alfredo Gulielmi, Serena Manfreda, Anna Rinaldi, Maria Novella Ringressi, Beatrice Brunoni, Giuseppe Salamone, Mirko Mangiapane, Paolino De Marco, Antonella La Brocca, Roberta Tutino, Vania Silvestri, Leo Licari, Tommaso Fontana, Nicolò Falco, Gianfranco Cocorullo, Mostafa Shalaby, Pierpaolo Sileri, Claudio Arcudi, Isam Bsisu, Khaled Aljboor, Lana Abusalem, Aseel Alnusairat, Ahmad Qaissieh, Emad Al‐Dakka, Ali Ababneh, Oday Halhouli, Taha Yusufali, Hussein Mohammed, Justus Lando, Robert Parker, Wairimu Ndegwa, Mantas Jokubauskas, Jolanta Gribauskaite, Donatas Venskutonis, Justas Kuliavas, Audrius Dulskas, Narimantas E. Samalavicius, Kristijonas Jasaitis, Audrius Parseliunas, Viktorija Nevieraite, Margarita Montrimaite, Evelina Slapelyte, Edvinas Dainius, Romualdas Riauka, Zilvinas Dambrauskas, Andrejus Subocius, Linas Venclauskas, Antanas Gulbinas, Saulius Bradulskis, Simona Kasputyte, Deimante Mikuckyte, Mindaugas Kiudelis, Justas Zilinskas, Tomas Jankus, Steponas Petrikenas, Matas Pažuskis, Zigmantas Urniežius, Mantas Vilčinskas, Vincas Jonas Banaitis, Vytautas Gaižauskas, Edvard Grisin, Povilas Mazrimas, Rokas Rackauskas, Mantas Drungilas, Karolis Lagunavicius, Vytautas Lipnickas, Dovilè Majauskyté, Valdemaras Jotautas, Tomas Abaliksta, Laimonas Uščinas, Gintaras Simutis, Adomas Ladukas, Donatas Danys, Erikas Laugzemys, Saulius Mikalauskas, Tomas Poskus, Elena Zdanyte Sruogiene, Petras Višinskas, Reda Žilinskienė, Deividas Dragatas, Andrius Burmistrovas, Zygimantas Tverskis, Arturas Vaicius, Ruta Mazelyte, Antanas Zadoroznas, Nerijus Kaselis, Greta Žiubrytė, Finaritra Casimir Fleur Prudence Rahantasoa, Luc Hervé Samison, Fanjandrainy Rasoaherinomenjanahary, Todisoa Emmanuella Christina Tolotra, Cornelius Mukuzunga, Vanessa Msosa, Chimwemwe Kwatiwani, Nelson Msiska, Feng Yih Chai, Siti Mohd Desa Asilah, Khuzaimah Zahid Syibrah, Pui Xin Chin, Afizah Salleh, Nur Zulaika Riswan, April Camilla Roslani, Hoong‐Yin Chong, Nora Abdul Aziz, Keat‐Seong Poh, Chu‐Ann Chai, Sandip Kumar, Mustafa Mohammed Taher, Nik Ritza Kosai, Dayang Nita Abdul Aziz, Reynu Rajan, Rokayah Julaihi, Durvesh Lacthman Jethwani, Muhammad Taqiyuddin Yahaya, Nik Azim Nik Abdullah, Susan Wndy Mathew, Kuet Jun Chung, Milaksh Kumar Nirumal, R. Goh Ern Tze, Syed Abdul Wahhab Eusoffee Wan Ali, Yiing Yee Gan, Jesse Ron Swire Ting, Samuel S. Y. Sii, Kean Leong Koay, Yi Koon Tan, Alvin Ee Zhiun Cheah, Chui Yee Wong, Tuan Nur'Azmah Tuan Mat, Crystal Yern Nee Chow, Prisca A.L. Har, Yishan Der, Yong Yong Tew, Fitjerald Henry, Xinwei Low, Ya Theng Neo, Hian Ee Heng, Shu Ning Kong, Cheewei Gan, Yi Ting Mok, Yee Wen Tan, Kandasami Palayan, Mahadevan Deva Tata, Yih Jeng Cheong, Kuhaendran Gunaseelan, Wan Nurul 'Ain Wan Mohd Nasir, Pigeneswaren Yoganathan, Eu Xian Lee, Jian Er Saw, Li Jing Yeang, Pei Ying Koh, Shyang Yee Lim, Shuang Yi Teo, Nicole Grech, Daniela Magri, Kristina Cassar, Christine Mizzi, Malcolm Falzon, Nihaal Shaikh, Ruth Scicluna, Stefan Zammit, Elaine Borg, Sean Mizzi, Svetlana Doris Brincat, Thelma Tembo, Vu Thanh Hien Le, Tara Grima, Keith Sammut, Kurt Carabott, Alexia Farrugia, Ciskje Zarb, Andre Navarro, Thea Dimech, Georgette Marie Camilleri, Isaac Bertuello, Jeffrey Dalli, Karl Bonavia, Samantha Corro‐Diaz, Marisol Manriquez‐Reyes, Antonio Ramos‐De la Medina, Amina Abdelhamid, Abdelmalek Hrora, Sarah Benammi, Houda Bachri, Meryem Abbouch, Khaoula Boukhal, Redouane Mammar Bennai, Abdelkader Belkouchi, Mohamed Sobhi Jabal, Chaymae Benyaiche, Maarten Vermaas, Lucia Duinhouwer, Javier Pastora, Greta Wood, Maria Soledad Merlo, Akinlabi Ajao, Omobolaji Ayandipo, Taiwo Lawal, Abdussemiu Abdurrazzaaq, Muslimat Alada, Abdulrasheed Nasir, James Adeniran, Olufemi Habeeb, Ademola Popoola, Ademola Adeyeye, Ademola Adebanjo, Opeoluwa Adesanya, Adewale Adeniyi, Henry Mendel, Bashir Bello, Umar Muktar, Adedapo Osinowo, Thomas Olagboyega Olajide, Oyindamola Oshati, George Ihediwa, Babajide Adenekan, Victor Nwinee, Felix Alakaloko, Adesoji Ademuyiwa, Olumide Elebute, Abdulrazzaq Lawal, Chris Bode, Mojolaoluwa Olugbemi, Alaba Adesina, Olubukola Faturoti, Oluwatomi Odutola, Oluwaseyi Adebola, Clement Onuoha, Ogechukwu Taiwo, Omolara Williams, Fatai Balogun, Olalekan Ajai, Mobolaji Oludara, Iloba Njokanma, Roland Osuoji, Stephen Kache, Jonathan Ajah, Jerry Makama, Ahmed Adamu, Suleiman Baba, Mohammad Aliyu, Shamsudeen Aliyu, Yahaya Ukwenya, Halima Aliyu, Tunde Sholadoye, Muhammad Daniyan, Oluseyi Ogunsua, Lofty‐John Anyanwu, Abdurrahaman Sheshe, Aminu Mohammad, Samson Olori, Philip Mshelbwala, Babatunde Odeyemi, Garba Samson, Oyediran Kehinde Timothy, Sani Ali Samuel, Anthony Ajiboye, Ademola Adeyeye, Isaac Amole, Olajide Abiola, Akin Olaolorun, Kjetil Søreide, Torhild Veen, Arezo Kanani, Kristian Styles, Ragnar Herikstad, Johannes Wiik Larsen, Jon Arne Søreide, Elisabeth Jensen, Mads Gran, Eirik Kjus Aahlin, Tina Gaarder, Peter Wiel Monrad‐Hansen, Pål Aksel Næss, Giedrius Lauzikas, Joachim Wiborg, Silje Holte, Knut Magne Augestad, Gurpreet Singh Banipal, Michela Monteleone, Thomas Tetens Moe, Johannes Kurt Schultz, Taher Al‐taher, Ayah Hamdan, Ayman Salman, Rana Saadeh, Aseel Musleh, Dana Jaradat, Soha Abushamleh, Sakhaa Hanoun, Amjad Abu Qumbos, Aseel Hamarshi, Ayman And Taher, Israa Qawasmi, Khalid Qurie, Marwa Altarayra, Mohammad Ghannam, Alaa Shaheen, Azher Herebat, Aram Abdelhaq, Ahmad Shalabi, Maram Abu‐toyour, Fatema Asi, Ala Shamasneh, Anwar Atiyeh, Mousa Mustafa, Rula Zaa'treh, Majd Dabboor, Enas Alaloul, Heba Baraka, Jehad Meqbil, Alaa Al‐Buhaisi, Mohamedraed Elshami, Samah Afana, Sahar Jaber, Said Alyacoubi, Yousef Abuowda, Tasneem Idress, Eman Abuqwaider, Sara Al‐saqqa, Alaa Bowabsak, Alaa El Jamassi, Doaa Hasanain, Hadeel Al‐farram, Maram Salah, Aya Firwana, Marwa Hamdan, Israa Awad, Ahmad Ashour, Fayez Elian Al Barrawi, Ahmed Al‐khatib, Maha Al‐faqawi, Mohamed Fares, Amjad Elmashala, Mohammad Adawi, Ihdaa Adawi, Reem Khreishi, Rose Khreishi, Ahmad ashour, Ahed Ghaben, Najwa Nadeem, Muhammad Saqlain, Jibran Abbasy, Abdul Rehman Alvi, Tanzeela Gala, Noman Shahzad, Kamran Faisal Bhopal, Zainab Iftikhar, Muhammad Talha Butt, Syed Asaat ul Razi, Asdaq Ahmed, Ali Khan Niazi, Ibrahim Raza, Fatima Baluch, Ahmed Raza, Ahmad Bani‐Sadar, Ahmad Uzair Qureshi, Muhammad Adil, Awais Raza, Mahnoor Javaid, Muhammad Waqar, Maryam Ali Khan, Mohammad Mohsin Arshad, Mohammadasim Amjad, Gustavo Miguel Machain Vega, Jorge Torres Cardozo, Marcelo O'Higgins Roche, Gustavo Rodolfo Pertersen Servin, Helmut Alfredo Segovia Lohse, Larissa Ines Páez Lopez, Ramón Augusto Melo Cardozo, Fernando Espinoza, Angel David Pérez Rojas, Diana Sanchez, Camila Sanchez Samaniego, Shalon Guevara Torres, Alexander Canta Calua, Cesar Razuri, Nadia Ortiz, Xianelle Rodriguez, Nahilia Carrasco, Fridiz Saravia, Hector Shibao Miyasato, María Valcarcel‐Saldaña, Ysabel Esthefany Alejos Bermúdez, Juan Carpio, Walter Ruiz Panez, Pedro Angel Toribio Orbegozo, Carolina Guzmán Dueñas, Kevin Turpo Espinoza, Ana Maria Sandoval Barrantes, Jorge Armando Chungui Bravo, Sebastian Shu, Lorena Fuentes‐Rivera, Carmen Fernández, Diego Romani, Bárbara Málaga, Joselyn Ye, Ricardo Velasquez, Jannin Salcedo, Ana Lucia Contreras‐Vergara, Angelica Genoveva Vergara Mejia, Maria Soledad Gonzales Montejo, Marilia Del Carmen Escalante Salas, Willy Alcca Ticona, Marvin Vargas, George Christian Manrique Sila, Robinson Mas, Arazzelly del Pilar Paucar, Armando José Román Velásquez, Alina Robledo‐Rabanal, Ludwing Alexander Zeta Solis, Kenny Turpo Espinoza, José Luis Hamasaki Hamaguchi, Erick Samuel Florez Farfan, Linda Alvi Madrid Barrientos, Juan Jaime Herrera Matta, John Jemuel V. Mora, Menold Archee P. Redota, Manuel Francisco Roxas, Maria Jesusa B. Maño, Marie Dione Parreno‐Sacdalan, Marie Carmela Lapitan, Christel Leanne Almanon, Maciej Walędziak, Rafał Roszkowski, Michał Janik, Anna Lasek, Piotr Major, Dorota Radkowiak, Mateusz Rubinkiewicz, Cristina Fernandes, Jose Costa‐Maia, Renato Melo, Liviu Muntean, Aurel Sandu Mironescu, Lucian Corneliu Vida, Amar Kourdouli, Mariuca Popa, Hogea Mircea, Mihaela Vartic, Bogdan Diaconescu, Matei Razvan Bratu, Ionut Negoi, Mircea Beuran, Cezar Ciubotaru, J.C Allen Ingabire, Alphonse Zeta Mutabazi, Norbert Uzabumwana, Dieudonne Duhoranenayo, Elio Jovine, Nicola Zanini, Giovanni Landolfo, Murad Aljiffry, Faisal Idris, Mohammed Saleh A. Alghamdi, Ashraf Maghrabi, Abdulmalik Altaf, Aroub Alkaaki, Ahmad Khoja, Abrar Nawawi, Sondos Turkustani, Eyad Khalifah, Ahmad Gudal, Adel Albiety, Sarah Sahel, Reham Alshareef, Mohammed Najjar Ahmed Alzahrani, Ahmed Alghamdi, Wedyan Alhazmi, Ghiath Al Saied, Mohammed Alamoudi, Muhammed Masood Riaz, Mazen Hassanain, Basmah Alhassan, Abdullah Altamimi, Reem Alyahya, Norah Al Subaie, Fatema Al Bastawis, Afnan Altamimi, Thamer Nouh, Roaa Khan, Milan Radojkovic, Ljiljana Jeremic, Milica Nestorovic, Jia Hao Law, Keith Say Kwang Tan, Ryan Choon Kiat Tan, Joel Kin Tan, Lau Wen Liang Joel, Bettina Lieske, Xue Wei Chan, Faith Qi Hui Leong, Choon Seng Chong, Sharon Koh, Kai Yin Lee, Kuok Chung Lee, Kent Pluke, Britta Dedekind, Puyearashid Nashidengo, Mark Ian Hampton, Johanna Joosten, Sanju Sobnach, Liana Roodt, Anthony Sander, James Pape, Richard Spence, Niveshni Maistry, Phumudzo Ndwambi, Kamau Kinandu, Myint Tun, Frederick Du Toit, Quinn Ellison, Sule Burger, DC Grobler, Lawrence Bongani Khulu, Rachel Moore, Vicky Jennings, Astrid Leusink, Nazmie Kariem, Juan Gouws, Kathryn Chu, Heather Bougard, Fazlin Noor, Angela Dell, Sarah Rayne, Stephanie Van Straten, Arvin Khamajeet, Serge Kapenda Tshisola, Kalangu Kabongo, Victor Kong, Yoshan Moodley, Frank Anderson, Thandinkosi Madiba, Flip du Plooy, Leila Hartford, Gareth Chilton, Parveen Karjiker, Matlou Ernest Mabitsela, Sibongile Ruth Ndlovu, Maria Badicel, Robert Jaich, Jaime Ruiz‐Tovar, Luis Garcia‐Florez, Jorge L. Otero‐Díez, Virginia Ramos Pérez, Nuria Aguado Suárez, Javier Minguez García, Sara Corral Moreno, Maria Vicenta Collado, Virginia Jiménez Carneros, Javier García Septiem, Mariana Gonzalez, Antonio Picardo, Enrique Esteban, Esther Ferrero, Irene Ortega, Eloy Espin‐Basany, Ruth Blanco‐Colino, Valeria Andriola, Lorena Solar García, Elisa Contreras, Carmen García Bernardo, Janet Pagnozzi, Sandra Sanz, Alberto Miyar de León, Asnel Dorismé, Joseluis Rodicio, Aida Suarez, Jessica Stuva, Tamara Diaz Vico, Laura Fernandez‐Vega, Carla Soldevila‐Verdeguer, Fatima Sena‐Ruiz, Natalia Pujol‐Cano, Paula Diaz‐Jover, José Maria Garcia‐Perez, Juan Jose Segura‐Sampedro, Cristina Pineño‐Flores, David Ambrona‐Zafra, Andrea Craus‐Miguel, Patricia Jimenez‐Morillas, Angela Mazzella, A.B Jayathilake, S.P.B Thalgaspitiya, L.S. Wijayarathna, P.M.S.N. Wimalge, Hakeem Ayomi Sanni, Aliyu Ndajiwo, Ogheneochuko Okenabirhie, Anmar Homeida, Abobaker Younis, Omer Abdelbagi Omer, Mustafa Abdulaziz, Ali Mussad, Ali Adam, Yucel Cengiz, Ida Björklund, Sandra Ahlqvist, Sandra Ahlqvist, Anders Thorell, Fredrik Wogensen, Arestis Sokratous, Michaela Breistrand, Hildur Thorarinsdottir, Johanna Sigurdadottir, Maziar Nikberg, Abbas Chabok, Maria Hjertberg, Peter Elbe, Deborah Saraste, Wiktor Rutkowski, Louise Forlin, Karoliina Niska, Malin Sund, Dennis Oswald, Georgios Peros, Rafael Bluelle, Katharina Reinisch, Daniel Frey, Adrian Palma, Dimitri Aristotle Raptis, Lucius Zumbühl, Markus Zuber, Roger Schmid, Gabriela Werder, Antonio Nocito, Alexandra Gerosa, Silke Mahanty, Lukas Werner Widmer, Julia Müller, Alissa Gübeli, Grzegorz Zuk, Osman Bilgin Gulcicek, Yuksel Altinel, Talar Vartanoglu, Emin Kose, Servet Rustu Karahan, Mehmet Can Aydin, Nuri Alper Sahbaz, Ilkay Halicioglu, Halil Alis, Ipek Sapci, Can Adıyaman, Ahmet Murat Pektaş, Turgut Bora Cengiz, Ilkan Tansoker, Vedatcan Işler, Muazzez Cevik, Deniz Mutlu, Volkan Ozben, Berk Baris Ozmen, Sefa Bayram, Sinem Yolcu, Berna Buse Kobal, Ömer Faruk Toto, Haluk Cem Çakaloğlu, Kagan Karabulut, Vahit Mutlu, Bahar Busra Ozkan, Saban Celik, Anil Semiz, Selim Bodur, Enisburak Gül, Busra Murutoglu, Reyyan Yildirim, Bahadir Emre Baki, Ekin Arslan, Mehmet Ulusahin, Ali Guner, Nathan Walker, Nikhita Shrimanker, Michael Stoddart, Simon Cole, Ryan Breslin, Ravi Srinivasan, Mohamed Elshaer, Kristina Hunter, Ahmed Al‐Bahrani, Ignatius Liew, Nora Grace Mairs, Alistair Rocke, Lachlan Dick, Mobeen Qureshi, Debkumar Chowdhury, Naomi Wright, Clare Skerritt, Dorothy Kufeji, Adrienne Ho, Tharindra Dissanayake, Athula Tennakoon, Wadah Ali, Shujing Jane Lim, Charlene Tan, Stephen O'Neill, Catrin Jones, Stephen Knight, Dima Nassif, Abhishek Sharma, Oliver Warren, Rebecca White, Aia Mehdi, Nathan Post, Eliana Kalakouti, Enkhbat Dashnyam, Frederick Stourton, Ioannis Mykoniatis, Chelise Currow, Francisca Wong, Ashish Gupta, Veeranna Shatkar, Joshua Luck, Suraj Kadiwar, Alexander Smedley, Rebecca Wakefield, Philip Herrod, James Blackwell, Jonathan Lund, Fraser Cohen, Ashwath Bandi, Stefano Giuliani, Giles Bond‐Smith, Theodore Pezas, Neda Farhangmehr, Tomas Urbonas, Miklos Perenyei, Philip Ireland, Natalie Blencowe, Kirk Bowling, David Bunting, Lydia Longstaff, Neil Smart, Kenneth Keogh, Hyunjin Jeon, Muhammad Rafaih Iqbal, Shivun Khosla, Anna Jeffery, James Perera, Ella Teasdale, Ahmad Aboelkassem Ibrahem, Tariq Alhammali, Yahya Salama, Rakan Kabariti, Shaun Oram, Thomas Kidd, Fraser Cullen, Christopher Owen, Michael Wilson, Seehui Chiu, Hannah Sarafilovic, Jennifer Ploski, Elizabeth Evans, Athar Abbas, Sylvia Kamya, Norzawani Ishak, Carly Bisset, Cedar Andress, Ye Ru Chin, Priya Patel, David Evans, Anna Jeffery, James Perera, Aidan Haslegrave, Adam Boggon, Kirsten Laurie, Katie Connor, Thomas Mann, Dmitri Nepogodiev, Anahita Mansuri, Rachel Davies, Ewen Griffiths, Aized Raza Shahbaz, Calvin Eng, Farhat Din, Ariadne L'Heveder, Esther H.G. Park, Ramanish Ravishankar, Kirsten McIntosh, Jih Dar Yau, Luke Chan, Susan McGarvie, Lingshan Tang, Hui Lim, Suhhuey Yap, Jay Park, Zhan Herr Ng, Shahrukh Mirza, Yun Lin Ang, Luke Walls, Ella Teasdale, Chloe Roy, Simon Paterson‐Brown, Julian Camilleri‐Brennan, Kenneth Mclean, Michelle S D'Souza, Savva Pronin, David Ewart Henshall, Eunice Zuling Ter, Dina Fouad, Ashish Minocha, William English, Catrin Morgan, Dominic Townsend, Laura Maciejec, Shareef Mahdi, Onyinye Akpenyi, Elisabeth Hall, Hanaan Caydiid, Zakaria Rob, Tom Abbott, Hew D Torrance, Gareth Irwin, Robin Johnston, Mohammed Akil Gani, Gianpiero Gravante, Shivanchan Rajmohan, Kiran Majid, Shiva Dindyal, Christopher Smith, Madanmohan Palliyil, Sanjay Patel, Luke Nicholson, Neil Harvey, Katie Baillie, Sam Shillito, Suzanne Kershaw, Rebecca Bamford, Peter Orton, Elke Reunis, Robert Tyler, Wai Cheong Soon, Guled M. Jama, Dharminder Dhillon, Khyati Patel, Shayanthan Nanthakumaran, Rachel Heard, Kar Yan Chen, Behrad Barmayehvar, Uttaran Datta, Sivesh K Kamarajah, Sharad Karandikar, Sobhana Iftekhar Tani, Eimear Monaghan, Philippa Donnelly, Michael Walker, Jehangirshaw Parakh, Sarah Blacker, Anil Kaul, Arjun Paramasivan, Sameh Farag, Ashrafun Nessa, Salwa Awadallah, Jieqi Lim, James Chean Khun Ng, Katherine Gash, Ravi P. Kiran, Alice Murray, Eric Etchill, Mohini Dasari, Juan Puyana, Nadeem Haddad, Martin Zielinski, Asad Choudhry, Celeste Caliman, Mieshia Beamon, Therese Duane, Ragavan Narayanan, Mamta Swaroop, Jonathan Myers, Rebecca Deal, Erik Schadde, Mark Hemmila, Lena Napolitano, Kathleen To, Alex Makupe, Joseph Musowoya, Mayaba Maimbo, Niels Van Der Naald, Dayson Kumwenda, Alex Reece‐Smith, Kars Otten, Anna Verbeek, Marloes Prins, Data validators (GlobalSurg‐2), Alibeth Andres Baquero Suarez, Ruben Balmaceda, Chelsea Deane, Emilio Dijan, Mahmoud Elfiky, Laura Koskenvuo, Aurore Thollot, Bernard Limoges, Carmen Capito, Challine Alexandre, Henri Kotobi, Julien Leroux, Julien Rod, Kalitha Pinnagoda, Nicolas Henric, Olivier Azzis, Olivier Rosello, Poddevin Francois, Sara Etienne, Philippe Buisson, Sophian Hmila, Joe‐Nat Clegg‐Lamptey, Osman Imoro, Owusu Emmanuel Abem, Paul Wondoh, Dimitrios Papageorgiou, Vasiliki Soulou, Sabrina Asturias, Lenin Peña, Basant Kumar, Donal B O'Connor, Alberto Realis Luc, Alfio Alessandro Russo, Andrea Ruzzenente, Antonio Taddei, Camilla Cona, Corrado Bottini, Giovanni Pascale, Giuseppe Rotunno, Leonardo Solaini, Marco Maria Pascale, Margherita Notarnicola, Mario Corbellino, Michele Sacco, Paolo Ubiali, Roberto Cautiero, Tommaso Bocchetti, Elena Muzio, Vania Guglielmo, Eugenio Morandi, Patrizio Mao, Emilia De Luca, Margherita Notarnicola, Farah Mahmoud Ali, Justas Žilinskas, Kestutis Strupas, Paulius Kondrotas, Robertas Baltrunas, Juozas Kutkevicius, Povilas Ignatavicius, Choy Ling Tan, Jia Yng Siaw, Sir Young Yam, Ling Wilson, Mohamed Rezal Abdul Aziz, John Bondin, Carmina Diaz Zorrilla, Anass Majbar, Danjuma Sale, Lawal Abdullahi, Olabisi Osagie, Omolara Faboya, Adedeji Fatuga, Agboola Taiwo, Emeka Nwabuoku, Marte Bliksøen, Zain Ali Khan, Jazmin Coronel, Cesar Miranda, Idelso Vasquez, Luis M. Helguero‐Santin, Jennifer Rickard, Aurel Mironescu, Adesina Adedeji, Saleh Alqahtani, Max Rath, Michael Van Niekerk, Modise Zacharia Koto, Roel Matos‐Puig, Leif Israelsson, Tobias Schuetz, Mahmut Arif Yuksek, Meric Mericliler, Mehmet Uluşahin, Bernhard Wolf, Cameron Fairfield, Guo Liang Yong, Katharine Whitehurst, Michael Wilson, Natalie Redgrave, Caroluce K Musyoka, James Olivier, Kathryn Lee, Michael Cox, Muhamed M H Farhan‐Alanie, Rory Callan, Chali Chibuye, Protocol translators (GlobalSurg‐2), Tebian Hassanein Ahmed Ali, Syrine Rekhis, Muna Rommaneh, Oday Halhouli, Zi Hao Sam, Jacky Hong Chieh Chen, Dylan Roi, Lawani Ismaïl, Vasileios Kalles, Francesco Pata, Gabriela Elisa Nita, Federico Coccolini, Luca Ansaloni, Thays Brunelli Pugliesi, Gabriel Pardo, Ruth Blanco, Eugenio Grasset Escobar

## Abstract

**Background:**

End colostomy rates following colorectal resection vary across institutions in high‐income settings, being influenced by patient, disease, surgeon and system factors. This study aimed to assess global variation in end colostomy rates after left‐sided colorectal resection.

**Methods:**

This study comprised an analysis of GlobalSurg‐1 and ‐2 international, prospective, observational cohort studies (2014, 2016), including consecutive adult patients undergoing elective or emergency left‐sided colorectal resection within discrete 2‐week windows. Countries were grouped into high‐, middle‐ and low‐income tertiles according to the United Nations Human Development Index (HDI). Factors associated with colostomy formation *versus* primary anastomosis were explored using a multilevel, multivariable logistic regression model.

**Results:**

In total, 1635 patients from 242 hospitals in 57 countries undergoing left‐sided colorectal resection were included: 113 (6·9 per cent) from low‐HDI, 254 (15·5 per cent) from middle‐HDI and 1268 (77·6 per cent) from high‐HDI countries. There was a higher proportion of patients with perforated disease (57·5, 40·9 and 35·4 per cent; *P* < 0·001) and subsequent use of end colostomy (52·2, 24·8 and 18·9 per cent; *P* < 0·001) in low‐ compared with middle‐ and high‐HDI settings. The association with colostomy use in low‐HDI settings persisted (odds ratio (OR) 3·20, 95 per cent c.i. 1·35 to 7·57; *P* = 0·008) after risk adjustment for malignant disease (OR 2·34, 1·65 to 3·32; *P* < 0·001), emergency surgery (OR 4·08, 2·73 to 6·10; *P* < 0·001), time to operation at least 48 h (OR 1·99, 1·28 to 3·09; *P* = 0·002) and disease perforation (OR 4·00, 2·81 to 5·69; *P* < 0·001).

**Conclusion:**

Global differences existed in the proportion of patients receiving end stomas after left‐sided colorectal resection based on income, which went beyond case mix alone.

## Graphical abstract

This secondary analysis of an international prospective multicentre observational study demonstrates significant variation in the proportion of patients receiving an end stoma after left‐sided colorectal resection between high‐ and low–middle‐income countries; this cannot be accounted for by case mix alone. This could relate to unmeasured patient risk characteristics, but may also reflect delayed access to surgical care, lack of equipment for anastomosis formation, or differences in subspecialist colorectal training.

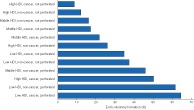

Wide variation in stoma rates

## Introduction

In 2015, the Lancet Commission on Global Surgery highlighted a substantial gap in access to safe and affordable surgical care across low‐ and middle‐income countries (LMICs), raising the priority of surgery on the global health agenda^1^. Despite this, reporting of outcomes following abdominal surgery from many LMICs remains unstandardized and of mixed quality. Where high‐quality evidence is available, a threefold higher risk of death in low‐ *versus* high‐income settings has been observed^2^. However, other key outcomes from the surgical management of colorectal cancer or benign colorectal disease in LMICs have been particularly poorly profiled to date^3^.

End colostomy rates following colorectal cancer resection vary substantially between centres in high‐income countries, ranging from 15 to 70 per cent^4^. This may reflect variations in case mix, as the decision to create an end colostomy rather than a primary restorative anastomosis is influenced by the urgency of presentation, the presence of operative field contamination, disease severity and stage, as well as functional status of the pelvic floor. For patients, quality of life with an end colostomy is influenced by multiple factors, including functional status, social support, income level, education and availability of specialist services^5^. The care requirements of a stoma may present a different psychosocial and physiological burden for patients in LMICs compared with those in high‐income settings. For example, geographical barriers and limited health resources are likely to raise treatment costs and reduce access to specialist equipment and services^6^, increasing the risk of catastrophic expenditure following colorectal surgery^7^. Examining international practice in stoma formation is therefore important in seeking to identify areas of variation and improve outcomes.

The primary aim of this study was to determine variation in rates of end colostomy formation following colorectal resection between low‐, middle‐ and high‐Human Development Index (HDI) strata, after adjusting for patient, disease and operative factors. Secondary aims were to report the mode of presentation, rate of laparoscopic surgery, and to determine any relationship between stoma formation and postoperative mortality in patients undergoing resections.

## Methods

### Protocol and network

This study was an exploratory subgroup analysis from two international, multicentre, prospective cohort studies conducted according to previously published protocols (NCT02179112, NCT02662231)^2^, ^8^. These protocols were disseminated through social media, and national and international surgical and anaesthetic associations. Briefly, the model required small teams of local investigators to collect data on prospectively determined items, coordinated by regional and national lead investigators, across short time windows, with pooled analysis by a central steering committee.

### Patients and settings

Any hospital providing both emergency surgery and elective colorectal surgical services was eligible to contribute patients to this study. Patients were included during at least one discrete 2‐week period between 1 July 2014 and 31 December 2014 (GlobalSurg‐1) and 4 January 2016 and 31 July 2016 (GlobalSurg‐2). To maximize inclusiveness and minimize burden on resource‐constrained clinicians, collaborators were permitted to collect data within any 2‐week interval across this time window, so long as data collection was consecutive and case ascertainment was complete. Adult patients (aged over 16 years) undergoing elective (GlobalSurg‐2) or emergency (GlobalSurg‐1 and ‐2) left hemicolectomy, sigmoid colectomy or rectal resection were included. Emergency procedures were defined as unplanned operations occurring within 2 weeks of hospital admission, and included procedures for trauma and reoperation following surgical complications. Open, laparoscopic and laparoscopic converted to open procedures were all eligible. To reduce risk of bias based on case mix, only colorectal resections for a primary gastrointestinal indication were included. Patients were excluded if the primary indication for surgery was vascular, gynaecological, obstetric, urological or transplantation, or if they were undergoing multivisceral resection.

### Ethics and reporting

A UK National Health Service (NHS) Research Ethics review considered both studies exempt from formal research registration (South East Scotland Research Ethics Service, references NR/1404AB12 and NR/1510AB5). Individual centres were responsible for audit or institutional review board or ethical approval if required by local regulations. This study is reported according to the STROBE guidelines^9^.

### Outcome measures

The primary outcome measure was the end colostomy formation rate, defined as formation of an end colostomy during the index procedure without restorative anastomosis. The secondary outcome measure was the postoperative mortality rate (death within 30 days of the index procedure).

### Other included explanatory variables

Data variables were designed to be assessed objectively, standardizable and internationally relevant. Variables deemed candidates in the causal pathway for stoma formation were indication for surgery, urgency of surgery (elective/planned or emergency/unplanned (within 2 weeks of hospital admission)) and colonic or rectal perforation noted at the time of surgery. Variables deemed to be confounders associated with both the causal pathway and outcome measures included age, sex, ASA fitness classification, smoking status, use of the WHO Surgical Safety checklist^10^, and use of laparoscopic surgery.

### Data capture and validation

Study data were collected and managed using REDCap (Research Electronic Data Capture) tools hosted at the University of Edinburgh (https://www.project-redcap.org/). REDCap is a secure, web‐based application designed to support data capture for research studies, providing: an intuitive interface for validated data entry; audit trails for tracking data manipulation and export procedures; automated export procedures for seamless data downloads to common statistical packages; and procedures for importing data from external sources. In both studies, a local lead investigator was responsible for overall quality assurance, case ascertainment and data accuracy at each centre. Where missing data were identified, the lead investigator was contacted and asked to ensure completeness. Records from centres that had an overall data completion rate of less than 95 per cent were removed from this analysis.

### Statistical analysis

Variation across different international health settings was assessed by stratifying participating centres by country into tertiles according to HDI. This is a composite statistic of life expectancy, education and income indices published by the United Nations (http://hdr.undp.org/en/content/human-development-index-hdi). Differences between HDI tertiles were tested with the Pearson χ^2^ test and Kruskal–Wallis test for categorical and continuous variables respectively. Descriptive percentages are listed as low HDI *versus* middle HDI *versus* high HDI throughout for consistency. To account for case mix, mixed‐effects, hierarchical multilevel logistic regression models were constructed. Patients nested within countries were considered by a random‐effects model. Patient‐, disease‐ and operation‐specific variables considered *a priori* to be candidates in the causal pathway, or confounders to the included outcomes, were included and treated as fixed effects. Model residuals were checked at both levels, checking for first‐order interactions; these were included in final models if found to be influential. Final model selection was by minimizing the widely applicable information criterion (variables considered to be marginal candidates in the causal pathway, and that reduced the goodness of the model fit were removed). Any variables with an incident rate below 1 per cent were not taken forwards into the multivariable models. Model discriminative ability was determined using the C‐statistic (area under the receiver operator curve, AUC). Coefficients generated were presented as odds ratios (ORs) with 95 per cent confidence intervals. All analyses were performed using the R version 3.1.1 (R Foundation for Statistical Computing, Vienna, Austria) with packages forcats, tidyverse, Hmisc, ggplot2, scales, RColorBrewer, lme4, gmodels, pglm, summariser and pROC.

## Results

**Figure 1:**
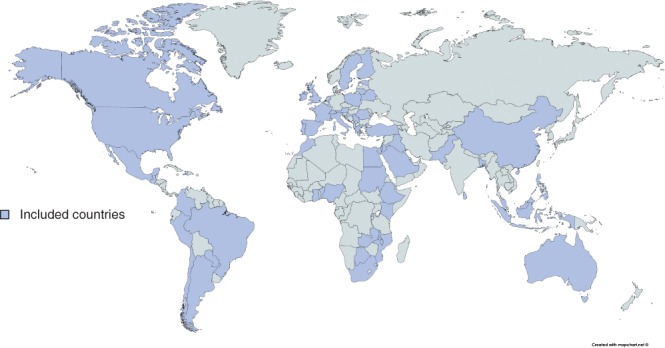
Map of included countries

**Figure 2:**
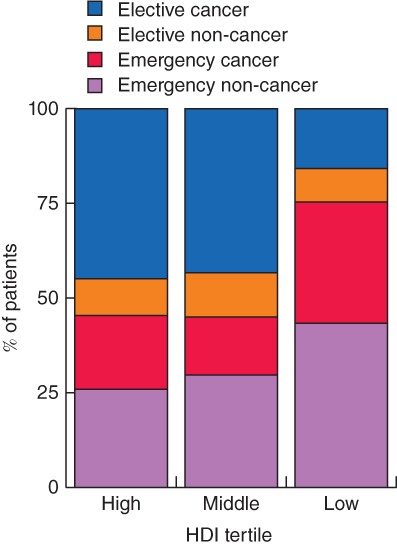
Presentation of patients undergoing left‐sided colorectal resection by Human Development Index tertile. HDI, Human Development Index

In total, 1635 patients from 242 hospitals in 57 countries (including 30 LMICs) undergoing left‐sided colorectal resection were included in this study (*Fig*. 1); 113 patients (6·9 per cent) were from low‐HDI, 254 (15·5 per cent) from middle‐HDI and 1268 (77·6 per cent) from high‐HDI countries. Patients from low‐ and middle‐HDI settings were significantly younger, more frequently men, lower risk (ASA grade below III) and less likely to smoke than those in high‐HDI settings (*Table* 1). Patients were more likely to present as an emergency in low‐HDI settings (low, 75·2 per cent; middle, 44·9 per cent; high, 45·5 per cent; *P* < 0·001) (*Fig*. 2) and more likely to have perforated disease at presentation (57·5, 40·9 and 35·4 per cent respectively; *P* < 0·001).

**Table 1:** Baseline demographics of patients undergoing left‐sided colorectal resection, grouped by Human Development Index tertile

	High HDI (*n* = 1268)	Middle HDI (*n* = 254)	Low HDI (*n* = 113)	*P* §
Age (years)*	65·9(13·8)	53·3(16·6)	51·4(16·9)	< 0·001¶
Sex				0·169
M	694 (54·7)	137 (53·9)	75 (66·4)	
F	533 (42·0)	107 (42·1)	36 (31·9)	
Missing	41 (3·2)	10 (3·9)	2 (1·8)	
ASA fitness grade				0·003
< III	706 (55·7)	170 (66·9)	70 (61·9)	
≥ III	553 (43·6)	80 (31·5)	41 (36·3)	
Missing	9 (0·7)	4 (1·6)	2 (1·8)	
Diabetes				0·133
No	1070 (84·4)	219 (86·2)	103 (91·2)	
Yes	198 (15·6)	35 (13·8)	10 (8·8)	
Smoking				0·026
No	884 (69·7)	181 (71·3)	94 (83·2)	
Yes	271 (21·4)	52 (20·5)	17 (15·0)	
Missing	113 (8·9)	21 (8·3)	2 (1·8)	
Malignancy				0·001
No	453 (35·7)	106 (41·7)	59 (52·2)	
Yes	815 (64·3)	148 (58·3)	54 (47·8)	
Urgency				< 0·001
Elective	691 (54·5)	140 (55·1)	28 (24·8)	
Emergency	577 (45·5)	114 (44·9)	85 (75·2)	
Time to operation (h)†				0·001
< 6	233 (18·4)	37 (14·6)	21 (18·6)	
6–11	89 (7·0)	22 (8·7)	16 (14·2)	
12–23	273 (21·5)	42 (16·5)	17 (15·0)	
24–47	272 (21·5)	39 (15·4)	19 (16·8)	
≥ 48	368 (29·0)	107 (42·1)	38 (33·6)	
Missing	33 (2·6)	7 (2·8)	2 (1·8)	
Laparoscopic				< 0·001
No	892 (70·3)	215 (84·6)	112 (99·1)	
Yes	376 (29·7)	39 (15·4)	1 (0·9)	
Perforated disease				< 0·001
No	813 (64·1)	147 (57·9)	47 (41·6)	
Yes	449 (35·4)	104 (40·9)	65 (57·5)	
Missing	6 (0·5)	3 (1·2)	1 (0·9)	
Checklist‡				< 0·001
No, not available	157 (12·4)	40 (15·7)	23 (20·4)	
No, but available	37 (2·9)	27 (10·6)	44 (38·9)	
Yes	1066 (84·1)	184 (72·4)	46 (40·7)	
Missing	8 (0·6)	3 (1·2)	0 (0)	

Values in parentheses are percentages by column, unless indicated otherwise;

* values are mean(s.d.).

† Time from presentation to index procedure.

‡ WHO Surgical Safety Checklist. HDI, Human Development Index.

§ Pearson χ^2^ test, except

¶ Kruskal–Wallis test.

**Figure 3:**
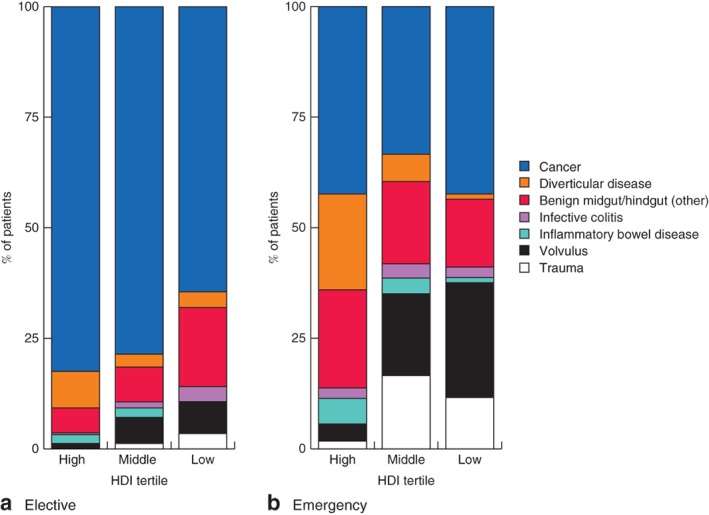
Indications for left‐sided colorectal resection by Human Development Index tertile and urgency of surgery. **a** Elective and **b** emergency. HDI, Human Development Index

Disease profiles in patients from low‐HDI settings were different from those in middle‐ and high‐HDI settings (*Fig*. 3). Fewer procedures were performed for malignancy (47·8, 58·3 and 64·3 per cent respectively; *P =* 0·001), diverticulitis (1·7, 4·3 and 14·2 per cent; *P* < 0·001) and inflammatory bowel disease (0, 1·6 and 1·4 per cent; *P =* 0·007), but a greater proportion of procedures were for volvulus (21·2, 7·5 and 2·4 per cent; *P* < 0·001) and trauma (9·7, 8·3 and 0·8 per cent respectively; *P* < 0·001). An overall delay from presentation to surgery of at least 48 h was more common in both low‐ and middle‐HDI than high‐HDI countries (33·6, 42·1 and 29·0 per cent; *P* < 0·001). A WHO checklist was used in only 40·7 per cent of operations in low‐HDI countries compared with 72·4 and 84·1 per cent in middle‐ and high‐HDI countries respectively. Half as many patients in middle‐HDI countries had a planned laparoscopic operation than in high‐HDI countries (15·4 *versus* 29·7 per cent; *P* < 0·001). Only one patient from a low‐HDI country had laparoscopic surgery (this was subsequently excluded from the mixed‐effects models).

### Variation in rates of end colostomy formation

**Figure 4:**
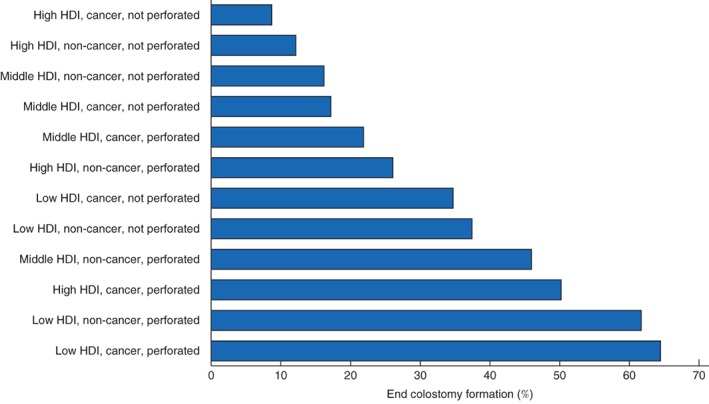
End colostomy formation rates by Human Development Index tertile, indication for surgery and presence of perforated disease. HDI, Human Development Index

Some 362 patients received an end colostomy (22·1 per cent) and 1273 a primary anastomosis (77·9 per cent) (*Table* 2). Of patients with an anastomosis, 211 (16·6 per cent) underwent left hemicolectomy, 40 (3·1 per cent) transverse or extended left hemicolectomy, 611 (48·0 per cent) sigmoid colectomy and 411 (32·3 per cent) rectal resection. Patients who received an end colostomy were more commonly high risk (ASA at least grade III: 48·9 *versus* 39·0 per cent; *P =* 0·004), had a benign indication (including trauma: 43·9 *versus* 36·1 per cent; *P =* 0·006) and perforated disease (66·6 *versus* 29·6 per cent; *P* < 0·001). Emergency surgery (77·1 *versus* 39·0 per cent; *P* < 0·001), open surgery (85·9 *versus* 71·3 per cent; *P* < 0·001) and a delay to surgery of 48 h or more (43·4 *versus* 28·0 per cent; *P* < 0·001) were also more common in the end colostomy group. Patients underwent formation of an end colostomy twice as frequently in low‐ compared with middle‐ or high‐HDI countries (52·2, 24·8 and 18·9 per cent; *P* < 0·001). *Fig*. 4 shows end colostomy formation rates across HDI strata, indications for surgery and the presence or absence of perforated disease.

**Table 2:** Baseline demographics of patients undergoing left‐sided colorectal resection, grouped by whether they underwent end colostomy formation or primary restorative anastomosis

	Anastomosis (*n* = 1273)	End colostomy (*n* = 362)	*P* §
HDI tertile			< 0·001
High	1028 (81·1)	240 (18·9)	
Middle	191 (75·2)	63 (24·8)	
Low	54 (47·8)	59 (52·2)	
Age (years)*	63·6(14·5)	60·5(18·4)	0·025¶
Sex			0·108
M	714 (78·8)	192 (21·2)	
F	513 (75·9)	163 (24·1)	
Missing	46 (87)	7 (13)	
ASA grade			0·004
< III	764 (80·8)	182 (19·2)	
≥ III	497 (73·7)	177 (26·3)	
Missing	12 (80)	3 (20)	
Diabetes			0·524
No	1080 (77·6)	312 (22·4)	
Yes	193 (79·4)	50 (20·6)	
Smoking			0·122
No	918 (79·2)	241 (20·8)	
Yes	253 (74·4)	87 (25·6)	
Missing	102 (75·0)	34 (25·0)	
Malignancy			0·006
No	459 (74·3)	159 (25·7)	
Yes	814 (80·0)	203 (20·0)	
Urgency			< 0·001
Elective	776 (90·3)	83 (9·7)	
Emergency	497 (64·0)	279 (36·0)	
Time to operation (h)†			< 0·001
< 6	230 (79·0)	61 (21·0)	
6–11	101 (79·5)	26 (20·5)	
12–23	283 (85·2)	49 (14·8)	
24–47	268 (81·2)	62 (18·8)	
≥ 48	356 (69·4)	157 (30·6)	
Missing	35 (83)	7 (17)	
Laparoscopic			< 0·001
No	908 (74·5)	311 (25·5)	
Yes	365 (87·7)	51 (12·3)	
Perforated disease			< 0·001
No	887 (88·1)	120 (11·9)	
Yes	377 (61·0)	241 (39·0)	
Missing	9 (90)	1 (10)	
Checklist‡			0·047
No, not available	178 (80·9)	42 (19·1)	
No, but available	73 (67·6)	35 (32·4)	
Yes	1013 (78·2)	283 (21·8)	
Missing	9 (82)	2 (18)	

Values in parentheses are percentages by row, unless indicated otherwise;

* values are mean(s.d.).

† Time from presentation to index procedure.

‡ WHO Surgical Safety Checklist. HDI, Human Development Index.

§ Pearson χ^2^ test, except

¶ Kruskal–Wallis test.

In univariable analysis, middle‐HDI (OR 1·41, 95 per cent c.i. 1·02 to 1·93; *P =* 0·033) and low‐HDI (OR 4·68, 3·15 to 6·96; *P* < 0·001) tertile were both strongly associated with end colostomy formation, as were ASA grade III or higher, malignancy, emergency surgery, a time to operation of 12–23 h or 48 h and over, perforated disease and absence of checklist use where it was available (*Table* 3). In the multilevel model, low‐HDI tertile retained an association with colostomy formation (OR 3·20, 1·35 to 7·57; *P =* 0·008), despite adjustment for malignant disease (OR 2·34, 1·65 to 3·32; *P* < 0·001), emergency surgery (OR 4·08, 2·73 to 6·10; *P* < 0·001), a time to operation of 48 h or longer (OR 1·99, 1·28 to 3·09; *P =* 0·002) and perforation (OR 4·00, 2·81 to 5·69; *P* < 0·001). The model demonstrated excellent discrimination (AUC 0·85) (*Table* 3).

**Table 3:** Factors associated with end colostomy formation in univariable and multilevel mixed‐effects logistic regression models

			Univariable analysis		Multilevel analysis	
	Anastomosis	End colostomy	Odds ratio*	*P*	Odds ratio*	*P*
HDI tertile						
High	1028 (80·8)	240 (66·3)	1·00 (reference)		1·00 (reference)	
Middle	191 (15·0)	63 (17·4)	1·41 (1·02, 1·93)	0·033	1·11 (0·53, 2·32)	0·777
Low	54 (4·2)	59 (16·3)	4·68 (3·15, 6·96)	< 0·001	3·20 (1·35, 7·57)	0·008
Age (years)	63·6(14·5)†	60·5(18·4)†	0·99 (0·98, 0·99)	0·001	0·99 (0·98, 1·00)	0·061
Sex						
M	714 (58·2)	192 (54·1)	1·00 (reference)		1·00 (reference)	
F	513 (41·8)	163 (45·9)	1·18 (0·93, 1·50)	0·169	1·17 (0·85, 1·59)	0·338
ASA fitness grade						
< III	764 (60·6)	182 (50·7)	1·00 (reference)		1·00 (reference)	
≥ III	497 (39·4)	177 (49·3)	1·49 (1·18, 1·89)	0·001	1·22 (0·87, 1·71)	0·256
Diabetes						
No	1080 (84·8)	312 (86·2)	1·00 (reference)		1·00 (reference)	
Yes	193 (15·2)	50 (13·8)	0·90 (0·64, 1·25)	0·525	1·08 (0·69, 1·68)	0·744
Smoking						
No	918 (78·4)	241 (73·5)	1·00 (reference)		1·00 (reference)	
Yes	253 (21·6)	87 (26·5)	1·31 (0·98, 1·73)	0·061	0·97 (0·68, 1·39)	0·889
Malignancy						
No	459 (36·1)	159 (43·9)	1·00 (reference)		1·00 (reference)	
Yes	814 (63·9)	203 (56·1)	0·72 (0·57, 0·91)	0·007	2·34 (1·65, 3·32)	< 0·001
Urgency						
Elective	776 (61·0)	83 (22·9)	1·00 (reference)		1·00 (reference)	
Emergency	497 (39·0)	279 (77·1)	5·25 (4·03, 6·91)	< 0·001	4·08 (2·73, 6·10)	< 0·001
Time to operation (h)‡						
< 6	230 (18·6)	61 (17·2)	1·00 (reference)		1·00 (reference)	
6–11	101 (8·2)	26 (7·3)	0·97 (0·57, 1·61)	0·910	0·65 (0·34, 1·23)	0·184
12–23	283 (22·9)	49 (13·8)	0·65 (0·43, 0·99)	0·044	0·76 (0·44, 1·29)	0·308
24–47	268 (21·6)	62 (17·5)	0·87 (0·59, 1·30)	0·498	1·24 (0·73, 2·11)	0·424
≥ 48	356 (28·8)	157 (44·2)	1·66 (1·19, 2·35)	0·003	1·99 (1·28, 3·09)	0·002
Laparoscopic§						
No	908 (71·3)	311 (85·9)	1·00 (reference)		–	
Yes	365 (28·7)	51 (14·1)	0·41 (0·29, 0·56)	< 0·001	–	
Perforated disease						
No	887 (70·2)	120 (33·2)	1·00 (reference)		1·00 (reference)	
Yes	377 (29·8)	241 (66·8)	4·73 (3·69, 6·08)	< 0·001	4·00 (2·81, 5·69)	< 0·001
Checklist¶						
No, not available	178 (14·1)	42 (11·7)	1·00 (reference)		1·00 (reference)	
No, but available	73 (5·8)	35 (9·7)	2·03 (1·20, 3·44)	0·008	1·10 (0·50, 2·41)	0·813
Yes	1013 (80·1)	283 (78·6)	1·18 (0·83, 1·72)	0·359	0·83 (0·44, 1·58)	0·576

Values in parentheses are percentages by column unless indicated otherwise;

* values in parentheses are 95 per cent confidence intervals and

† values are mean(s.d.).

‡ Time from presentation to index procedure.

§ Not included in multilevel model owing to low event rate in low‐Human Development Index (HDI) tertile (less than 1 per cent).

¶ WHO Surgical Safety Checklist.

### Variation in mortality

**Figure 5:**
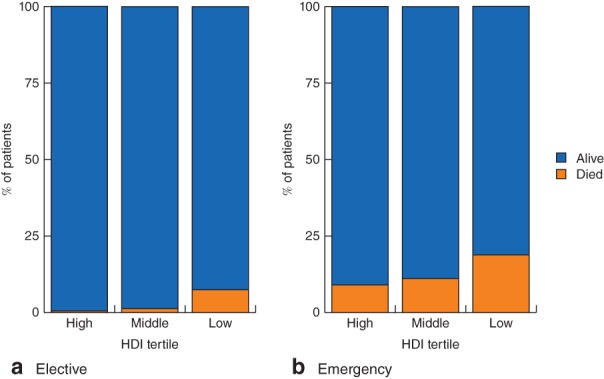
Percentage of patients who died within 30 days after left‐sided colorectal resection by Human Development Index tertile and urgency of surgery. **a** Elective and **b** emergency. HDI, Human Development Index

The unadjusted 30‐day postoperative mortality rates were three times higher in low‐HDI countries than in middle‐ and high‐HDI settings (15·9, 5·5 and 4·6 per cent respectively) (*Fig*. 5). Patients with an end colostomy had a significantly higher risk of death (adjusted OR 2·18, 95 per cent 1·23 to 3·85; *P =* 0·007), as did those from a low‐HDI tertile (OR 2·80, 1·00 to 7·82; *P =* 0·050), older patients, those with an ASA grade of at least III, patients having emergency surgery, and those with a delay to surgery of 24–47 h (*Table* 4). The benefit of use of the WHO Checklist in theatre reached borderline significance (OR 0·50, 0·22 to 1·13; *P =* 0·094). The model demonstrated excellent discrimination (AUC 0·89).

**Table 4:** Factors associated with mortality in patients undergoing left‐sided colorectal resection in univariable and multilevel, multivariable logistic regression models

			Univariable analysis		Multilevel analysis	
	Alive	Died	Odds ratio*	*P*	Odds ratio*	*P*
HDI tertile						
High	1200 (78·8)	58 (64)	1·00 (reference)		1·00 (reference)	
Middle	229 (15·0)	14 (16)	1·26 (0·67, 2·24)	0·443	1·60 (0·64, 3·97)	0·313
Low	93 (6·1)	18 (20)	4·00 (2·21, 6·95)	< 0·001	2·80 (1·00, 7·82)	0·050
Age (years)	62·7(15·3)†	69·1(15·9)†	1·03 (1·02, 1·05)		1·03 (1·01, 1·05)	0·001
Sex						
M	847 (57·5)	44 (51)	1·00 (reference)		1·00 (reference)	
F	625 (42·5)	43 (49)	1·32 (0·86, 2·04)	0·203	1·40 (0·82, 2·39)	0·214
ASA fitness grade						
< III	921 (61·0)	16 (18)	1·00 (reference)		1·00 (reference)	
≥ III	589 (39·0)	74 (82)	7·23 (4·29, 12·97)	< 0·001	6·16 (3·12, 12·19)	< 0·001
Diabetes						
No	1296 (85·2)	75 (83)	1·00 (reference)		1·00 (reference)	
Yes	226 (14·8)	15 (17)	1·15 (0·62, 1·98)	0·639	0·86 (0·43, 1·73)	0·681
Smoking						
No	1077 (77·3)	68 (78)	1·00 (reference)		1·00 (reference)	
Yes	316 (22·7)	19 (22)	0·95 (0·55, 1·58)	0·855	0·73 (0·39, 1·39)	0·345
Malignancy						
No	558 (36·7)	50 (56)	1·00 (reference)		1·00 (reference)	
Yes	964 (63·3)	40 (44)	0·46 (0·30, 0·71)	< 0·001	0·83 (0·48, 1·44)	0·503
Urgency						
Elective	837 (55·0)	9 (10)	1·00 (reference)		1·00 (reference)	
Emergency	685 (45·0)	81 (90)	11·00 (5·79, 23·68)	< 0·001	4·92 (2·18, 11·13)	< 0·001
Time to operation (h)‡						
< 6	267 (18·0)	18 (21)	1·00 (reference)		1·00 (reference)	
6–11	111 (7·5)	15 (17)	2·00 (0·96, 4·12)	0·058	1·12 (0·46, 2·72)	0·800
12–23	314 (21·2)	17 (19)	0·80 (0·40, 1·60)	0·529	0·98 (0·43, 2·19)	0·952
24–47	320 (21·6)	5 (6)	0·23 (0·08, 0·59)	0·004	0·21 (0·06, 0·70)	0·011
≥ 48	470 (31·7)	33 (38)	1·04 (0·58, 1·92)	0·893	0·78 (0·39, 1·59)	0·497
Laparoscopic§						
No	1115 (73·3)	85 (94)	1·00 (reference)		–	
Yes	407 (26·7)	5 (6)	0·16 (0·06, 0·36)	< 0·001	–	
Perforated disease						
No	961 (63·5)	32 (36)	1·00 (reference)		1·00 (reference)	
Yes	552 (36·5)	57 (64)	3·10 (2·00, 4·89)	< 0·001	1·07 (0·59, 1·92)	0·833
Checklist¶						
No, not available	197 (13·0)	17 (19)	1·00 (reference)		1·00 (reference)	
No, but available	92 (6·1)	12 (13)	1·51 (0·68, 3·27)	0·299	1·38 (0·46, 4·11)	0·564
Yes	1223 (80·9)	61 (68)	0·58 (0·34, 1·04)	0·054	0·50 (0·22, 1·13)	0·094
Anastomosis/colostomy						
Anastomosis	1208 (79·4)	48 (53)	1·00 (reference)		1·00 (reference)	
End colostomy	314 (20·6)	42 (47)	3·37 (2·18, 5·19)	< 0·001	2·18 (1·23, 3·85)	0·007

Values in parentheses are percentages by column unless indicated otherwise;

* values in parentheses are 95 per cent confidence intervals and

† values are mean(s.d.).

‡ Time from presentation to index procedure.

§ Not included in multilevel model owing to low event rate in low Human Development Index (HDI) tertile (less than 1 per cent).

¶ WHO Surgical Safety Checklist.

## Discussion

This study demonstrated that end stoma rates in low‐HDI countries were twice those in middle‐ and three times those in high‐HDI countries. As each of the HDI strata included multiple hospitals of different size and nature, it suggests that variation based on income per capita may be more important than variation within countries. The difference between groups is partly explained by differences in case mix, with greater emergency presentation of both malignant and non‐malignant conditions in low‐HDI settings. This association persisted despite adjustment, suggesting that other factors may contribute to this variation.

Patients in LMICs were more likely to present as emergencies and to have perforated disease than patients in high‐HDI settings. In part, this reflects differences in the overall disease burden, with trauma and volvulus being more common in LMICs. However, the increased frequency of emergency procedures for malignancy in LMICs may reflect barriers to accessing care and treatment for non‐communicable disease in LMICs^1^, ^3^. These may include limited implementation of screening programmes, inefficient referral pathways, the relatively high cost of investigations such as endoscopy^3^, ^11^, ^12^, as well as some patients having limited access to health education or a preference to seek care from traditional healers^13^, ^14^, ^15^, ^16^. The greater burden of emergency surgery suggests that patients in LMICs may be more likely to delay a decision to seek healthcare until they have deteriorated with complicated, advanced disease. Because significant populations live more than a 2‐h drive from the nearest hospital^17^, ^18^, patients' conditions may deteriorate further owing to delays while identifying affordable and efficient means of transport^19^, ^20^. In LMICs, once patients reach hospital, delayed and lack of appropriate investigations, staff shortages, erratic electric and water supplies, and insufficient funds to pay for care can limit and further delay surgery^21^. In the present study, patients in LMICs were more likely to experience significant in‐hospital delays. Consistent with previous studies^22^, ^23^, this was associated with end stoma formation. It should be noted in the present data, however, that in‐hospital delay (48 h or more) was not associated with an increased risk of death in the mixed‐effects model. This may reflect appropriate delay of surgical intervention (such as for preoperative optimization of an obstructing cancer) and appropriate rationalization of resources (the most unwell patients were prioritized for early access to theatre resources) across included hospitals. The three stages of delay in accessing acute care, in making a decision to travel to hospital, in travelling to hospital, and in hospital^24^, all contribute to patients in LMICs being more likely to present acutely unwell with complicated disease that makes primary restorative surgery challenging, and influencing the decision whether primary anastomosis or end colostomy is appropriate^25^.

Differences in training and provision of specialist colorectal surgery, and lack of available or affordable equipment for technically difficult anastomoses, could also affect stoma rates. With fewer patients presenting with operable colorectal cancer in many low‐HDI countries^3^, ^12^ and fewer formal training opportunities, access to subspecialist colorectal services is limited^3^, ^26^, ^27^. High baseline mortality rates^2^, inadequate provision of critical care support^28^, ^29^ and insufficient medicolegal protection^30^ may also promote risk‐averse practices. Stapling devices may be unaffordable for both patient and provider in many LMICs, meaning that only selected patients have access to these techniques^31^. Similarly, although laparoscopic colorectal resection was performed in middle‐HDI settings, it was uncommon. Lack of affordable laparoscopic equipment, variable provision of training and hospital‐level difficulties, such as a reliable electrical supply, remain barriers to minimal access surgery in LMIC settings^32^, despite potential for patient benefit^33^, ^34^.

The high mortality rate for both elective and emergency surgery reported in this study supports previous findings that patients have a higher risk of death following surgery in low‐HDI settings which cannot be accounted for by case mix alone^2^, ^35^. The present analysis showed that patients undergoing end stoma formation were at increased risk of death. Despite adjustment, this finding could represent a surrogate marker of disease severity where the highest‐risk patients are being selected to receive a stoma. In the present study, it was not possible to measure physiological markers of disease severity beyond ASA classification (such as hypotension, tachycardia, high lactate level or an end‐organ perfusion deficit) that could influence surgical decision‐making and outcomes.

This study has important limitations that could affect its generalizability. As it included a relatively low mean number of patients per centre in a ‘snapshot’ methodology, no analysis was performed at a per‐centre or per‐country level. Although only one‐quarter of patients in the data set were from LMICs, sites across 30 countries contributed data, bolstering external generalizability across LMIC settings. Data were collected across all HDI tertiles in both emergency (GlobalSurg‐1 and ‐2) and elective (GlobalSurg‐2) settings, and are relevant to both planned and unplanned left‐sided colorectal resections, but numbers in some groups (such as elective operations for cancer in low‐HDI settings) were small. Further validation of these findings is therefore required in future work. Although there were no centre‐level exclusion criteria for case volume or infrastructure, a sampling bias is likely to exist, wherein the best resourced and/or academically affiliated centres within LMICs were more likely to access the study protocol and provide patient data than those in remote and rural settings. This may have led to an underestimate of the true rate of end stoma formation within LMICs.

Reported end colostomy rates have varied from 0 to as high as 74 per cent^25^, ^36^, ^37^, ^38^, ^39^ in groups including emergency surgery^39^, late presentations of cancer^25^, complications of infectious disease^38^ and traumatic injury^36^. The collaborative methodology in the present study enabled clinicians to enter data into a secure online platform contemporaneously alongside their clinical practice, in accordance with a prespecified protocol. This led to high levels of data accuracy and completeness^40^ and has provided the basis on which further studies can be developed to examine other factors that influence outcomes in different settings.

**Patient viewpoint**

This study reveals global variation in end colostomy rates after left‐sided colorectal resection; stoma rates in low‐HDI countries were twice those in middle‐ and three times those in high‐HDI countries.

Awakening after surgery with a colostomy will have been a traumatic experience for all 362 patients. I wish we could ask everyone who still survives today some honest questions about their quality of life since. I imagine those in high‐HDI countries will have adapted better to their changed bodies and altered selves than their low‐HDI counterparts.

In high‐HDI England my own stoma is easy to accommodate thanks to freely accessible healthcare, uninterrupted supplies of decent ileostomy bags, sanitation, plentiful water, an angel of a specialist stoma nurse, and legal protection from societal or workplace discrimination: I am fortunate to enjoy a lovely life as a ‘Bag Lady’.

The absence of such enabling factors can, however, make having a stoma far more burdensome in low‐HDI countries. Financial ruin, inability to resume usual daily activities, societal rejection, family/community shame, and becoming unemployable and unmarriageable are, sadly, common sequelae. Indeed, my East African‐born parents insist that had I not been ‘Made in Britain’ long after they relocated to England, I would have suffered ‘intolerable strife or loss of life’.

There is a real need to reduce avoidable stoma formation globally. This need is most pressing in low‐HDI countries where physical, psychological, economic, educational and social challenges are magnified. The insurmountable obstacles they may face in low‐HDI settings can lead patients to question whether surviving surgery is in fact the superior of the two possible outcomes. Thus, although surgeons in restricted‐resource settings may have good reason to fear the consequences of anastomotic leaks, patients may have greater reason to fear the lifelong consequences of a stoma.

 

Ms Azmina Verjee

*GlobalSurg UK Patient Representative*

## Supporting information


**Appendix S1** Collaborators
